# Molecular Dynamics of Artificially Pair-Decoupled Systems: An Accurate Tool for Investigating the Importance of Intramolecular Couplings

**DOI:** 10.1021/acs.jctc.3c00553

**Published:** 2023-09-12

**Authors:** Michele Gandolfi, Michele Ceotto

**Affiliations:** Dipartimento di Chimica, Università degli Studi di Milano, via Golgi 19, 20133 Milano, Italy

## Abstract

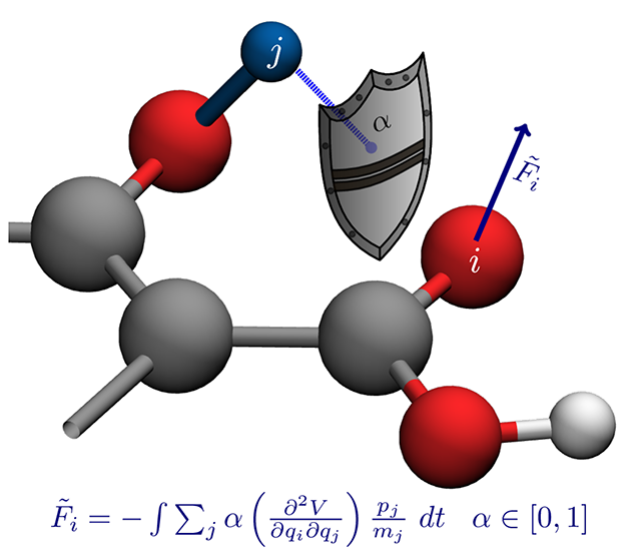

We propose a numerical technique to accurately simulate the vibrations of organic molecules in the gas phase, when pairs of atoms (or, in general, groups of degrees of freedom) are artificially decoupled, so that their motion is instantaneously decorrelated. The numerical technique we have developed is a symplectic integration algorithm that never requires computation of the force but requires estimates of the Hessian matrix. The theory we present to support our technique postulates a pair-decoupling Hamiltonian function, which parametrically depends on a decoupling coefficient α ∈ [0, 1]. The closer α is to 0, the more decoupled the selected atoms. We test the correctness of our numerical method on small molecular systems, and we apply it to study the vibrational spectroscopic features of salicylic acid at the Density Functional Theory *ab initio* level on a fitted potential. Our pair-decoupled simulations of salicylic acid show that decoupling hydrogen-bonded atoms do not significantly influence the frequencies of stretching modes, but enhance enormously the out-of-plane wagging and twisting motions of the hydroxyl and carboxyl groups to the point that the carboxyl and hydroxyl groups may overcome high potential energy barriers and change the salicylic acid conformation after a short simulation time. In addition, we found that the acidity of salicylic acid is more influenced by the dynamical couplings of the proton of the carboxylic group with the carbon ring than with the hydroxyl group.

## Introduction

The coupling among atoms in molecular systems is a key concept in chemistry and materials science, especially in organic chemistry, where it allows for an intuitive and qualitative description of the rich reactivity of organic compounds. Direct evidence of the vibrational couplings can be observed in the features of the vibrational (IR and Raman) spectrum, and it can be measured^1−3^ in the off-diagonal features of 2D vibrational spectra. In theoretical chemistry, 2D vibrational spectra can be calculated either by using a model coupling Hamiltonian,^1,2^ semiclassical approaches,^3^ or other trajectory based methods.^4^ This field of research is important even outside the realm of spectroscopy, because rationalization of the vibrational spectra allows the prediction, for instance, of selectivity^5,6^ and reaction yields.^7^

The correlated/coupled motion of the nuclei in a molecular system is an ultrafast phenomenon, and, as such, it must be studied using either experimental ultrafast techniques, which include pulse probe methods, or computer simulation methods aimed at the interpretation and simulation of two-dimensional spectra.^2,8−10^ In theoretical chemistry, the identification of the uncoupled degrees of freedom is useful for computational methodologies that calculate the vibrational spectrum in reduced dimensionality, such as, for instance, semiclassical approaches,^11−22^ QM/MM calculations,^23^ tensor-trains and sum of products of basis functions methods^24−26^ and also the Multi-Configuration Time-Dependent Hartree method (MCTDH)^27−29^ and methods based on MCTDH-like ansatz.^30^ Applications of all the aforementioned methods imply either that part of a system is partially independent of another or that the two parts have an artificial interaction. Either way, there is no rigorous method to establish whether the approximation implied is appropriate or not.

A field of research that developed accurate techniques for investigating the couplings is the study of Intramolecular Vibrational energy Redistribution. (IVR)^31−33^ An accurate (yet expensive) procedure used to investigate normal mode IVR is the instantaneous normal-mode analysis,^32,34,35^ which consists in a rediagonalization of the nonequilibrium Hessian matrix, whose eigenvectors can then be reinterpreted in terms of the traditional normal modes. The main disadvantage of such a technique, apart from the frequent need for the second derivative matrix of the potential, is that it can investigate the couplings only in normal mode coordinates.

The aim of this study is to investigate the effects of the couplings from a *dynamical* perspective, accounting for the real time vibrations of the molecules and employing the intuitive Cartesian coordinate system. To that end we provide an entirely new approach to the study of couplings: We rely on a practical description of the coupling between pairs of degrees of freedom that, in its simplicity, allows us to define numerical experiments of artificially *de*coupled atoms in molecules. Specifically, we introduce a method that allows a real time, full dimensional, and numerically accurate simulation of an artificially decoupled system.

**Figure 1:**
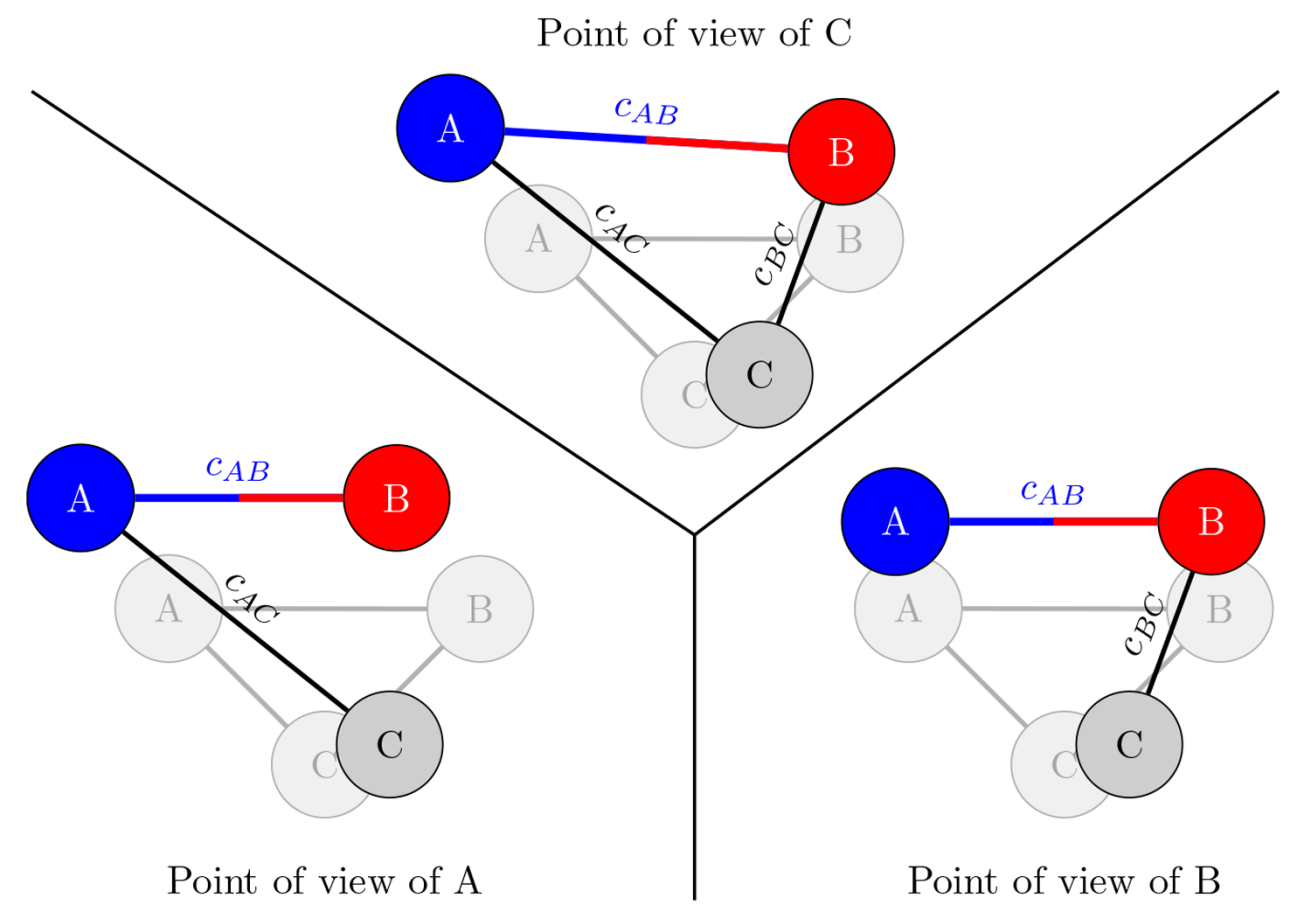
Cartoon of the pair-decoupling idea, representing the points of view of the three atoms compared to their initial geometry (the shaded molecules in the background). The top panel represents a snapshot of the simulation, which corresponds to the (objective) point of view of atom *C*. The left and right panels represent the points of view of the decoupled (α = 0) atoms *A* and *B*, respectively, in which either atom *Bperceives* atom *A* as if it never displaced (from its point of view) or vice versa. From the points of view of *A* and *B*, connector *c*_*AB*_ is set at the initial geometry value.

We define the atom–atom coupling as the phenomenon in which the force perceived by atom *A* depends on the position of atom *B*. In such cases, we would say that the motion of *A* and *B* is correlated or that *A* and *B* are coupled. First of all, we imagine a molecule represented by a collection of atoms, in which *each* pair of atoms are linked by the end points of a connector. If we put the molecule in its geometrical equilibrium and then abruptly displace a single atom, we would have a force acting on every atom that tries to move the whole system toward the nearest potential minimum. The force acts by either elongating or compressing (compared to their state at equilibrium) each connector and trying to adjust it in response to the deformation. This means that atoms *A* and *B*, connected by the connector *c*_*AB*_, would feel either an attractive force that pulls them together or a repulsive one that pushes them apart. However, if we cheat the physics and artificially set this force to its value before the deformation, *A* and *B* would perceive each other just as if the system were still in equilibrium, and so the connector *c*_*AB*_ would not respond to the deformation, while all the other atoms and connectors would perceive the force and respond accordingly. For instance, another atom *C* would perceive the deformation, and *c*_*AB*_ might either elongate or compress (because of the compression and elongation of *c*_*AC*_ and/or *c*_*BC*_). As a result of this artificial intervention, the motion of decoupled atoms (*A* and *B*) in response to the deformation is directly uncorrelated (although it could be indirectly correlated via a third atom). A cartoon of the pair-decoupling idea is shown in Figure 1.

In practice, if *ḟ*_*AB*_ is the rate of change of the force perceived by the connector *c*_*AB*_ in normal conditions, we could artificially scale it as *ḟ*_*AB*_·α, where α is a real number between 0 and 1. Of course, the closer α is to 1, the more coupled *A* and *B* are and the more realistic the simulation is. On the contrary, the closer α is to 0, the more artificial it is. The practice of artificially modifying the potential is commonly used in accelerated molecular dynamics methods,^36−39^ to explore the configuration space faster.

We propose to perform the artificial decoupling in a molecular dynamics simulation, in which the atoms are moved according to their initial velocities and in which we use α to shield the atoms from seeing each other’s displacements. To reach this goal, we developed a very simple numerical technique, called the Symplectic Explicit with Force (SEF) integration algorithm that allows an accurate time evolution of pair-decoupled systems. Moreover, we show that the SEF integration of the equations of motion preserves the symplectic symmetry and preserves in a significant amount also time-reversibility. We also show how the time-reversibility and energy conservation properties are exact for harmonic potentials and still accurate when the potential is anharmonic. The reader should notice that the potential between the atoms *A* and *B* is not modified when they are in their equilibrium position: the attractive/repulsive contribution of the force on the pair is artificially modified only when they are displaced during time evolution. In fact, when α = 0, the pair-decoupling implies that pairs of atoms may perceive each other as if they remain in their initial position. This detail also implies that while the equilibrium properties of the system may be unaltered by the decoupling (as it can be the case of normal mode coordinate decoupling), the dynamical properties may change very significantly, as we shall see in the section dedicated to the salicylic acid decouplings.

Hereafter, the Theory and Methods section formalizes the pair-decoupling idea and presents the molecular dynamics modified integrator for artificially decoupled pairs of atoms. The Results section presents the benchmarking of the method and its application to salicylic acid. The Discussion and Conclusions section concludes the paper.

## Theory and Methods

### Decoupling Hamiltonian

As anticipated above, we describe molecular systems as composed of some arbitrarily pair-decoupled atoms. We call *H* = *K* + *V* the Hamiltonian of the fully coupled (normal) system and *H̃* = *K* + *Ṽ* the Hamiltonian of the corresponding pair-decoupled system. For the rest of the paper, we will assume that *V* (and *Ṽ*) contains the electron–electron, electron–nucleus, and nucleus–nucleus Coulomb interactions, as well as the electron exchange potential and the electronic kinetic energy. Thus, *V* (and *Ṽ*) is a function of the nuclei positions in the Born–Oppenheimer approximation, and *K* is the corresponding nuclear kinetic energy. We make the further approximation that the nuclei behave as classical particles so that we can express the classical pair-decoupled Hamiltonian as *H̃* = ∑_α_(*p̃*_α_^2^/2*m*_α_) + *Ṽ*(*q̃*), where we assign the tilde (∼) symbol to the canonical coordinates *q̃* and *p̃* to specify that they are phase-space coordinates of the pair-decoupled Hamiltonian. While we do not have an explicit expression for *Ṽ* in terms of *V*, we express the relationship in terms of the potential derivatives because we employ these for integrating the equation of motion. Specifically, we assume that the main coupling between pairs is given by the second order derivative terms with respect to coordinates *q*_*i*_ and *q*_*j*_ (i.e., the Hessian matrix elements *h*_*ij*_) and that it can be artificially scaled:
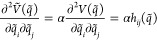
1

In eq 1, we have assumed that the decorrelation is for pairs of degrees of freedom, and α is the amount of decoupling, ranging from 1 (no decoupling) to 0 (fully decoupled). However, one can decouple multiple degrees of freedom at the same time. For instance, the pair-decoupled Hessian matrix for a three degrees of freedom system where two of them are fully coupled and the third one is partially decoupled from them is
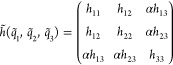
2

Notice that when α = 0, eq 2 corresponds to the Hessian matrix of two independent systems, one of which is two-dimensional and the second is monodimensional. When the time-evolution algorithm described in the next sections is applied to such a system, the evolution of the system is artificially separable, and the potential is up to the second order of the type *V* (*q̃*_1_, *q̃*_2_, *q̃*_3_) = *V*_1,2_(*q̃*_1_, *q̃*_2_) + *V*_3_(*q̃*_3_). Notice that in case of a truly separable potential, we could write *V*_3_(*q̃*_3_) – *V*_3_(*q̃*_3_^*eq*^) = *V* (*q̃*_1_, *q̃*_2_, *q̃*_3_) – *V* (*q̃*_1_, *q̃*_2_, *q̃*_3_^*eq*^) and *V*_1,2_(*q̃*_1_, *q̃*_2_) – *V*_1,2_(*q̃*_1_^*eq*^, *q̃*_2_^*eq*^) = *V* (*q̃*_1_, *q̃*_2_, *q̃*_3_) – *V* (*q̃*_1_^*eq*^, *q̃*_2_^*eq*^, *q̃*_3_). These last expressions correspond to the “projected potentials” used to compute the vibrational spectroscopic features of molecules as large as G-quadruplex in solution^40^ with the Divide-and-Conquer SemiClassical Initial Value Representation (DC-SCIVR) method.^14,15,20^ While the DC-SCIVR method simulates the dynamics of a system under a full dimensional potential and then approximates the classical action with a potential projected into subspaces,^14^ the algorithm we are presenting here evolves the dynamics entirely under the subspace-projected potential (or partially projected, when α ≠ 0). Furthermore, the decoupling could be applied to all the degrees of freedom pertaining to two atoms, that is, for instance, to the Cartesian product (*x*_1_, *y*_1_, *z*_1_) × (*x*_2_, *y*_2_, *z*_2_), to decouple atoms 1 and 2. In this paper, we focus on this Cartesian atom-decoupling scheme, as it appeals to chemical intuition, and we believe it would result as the most interesting for the chemical community. Nonetheless the decoupling idea could be applied to any coordinate system.

In practice, we propose a time-propagation rule that allows us to enforce the pair decoupling idea by making explicit the contributions to the force given by the Hessian matrix. In other words, we obtain the molecular dynamics force by numerically integrating the Hessian matrix over time as . However, this procedure is tricky because the variables *q̃*(*t*), *p̃*(*t*), and *F̃*(*t*) must be evaluated at the same time for the propagation to take place:
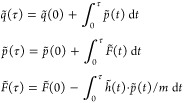
3where *h̃*(*t*) ≔ *α∂*^2^*V* (*q̃*(*t*))/*∂q̃*_*i*_*∂q̃*_*j*_ is the pair-decoupled Hessian matrix at time *t*. The procedure we have developed employs a standard symplectic integration for the coordinates *q̃* and *p̃*, while the force is updated from a time-integration of the Hessian matrix embedded with the symplectic map, consistent with the canonical variables. Specifically, for an integration that is accurate to order *n*, the practical update is a cycle over the integer *k*, up until *k* = *n* = 2 or 4 of the following four simple steps:
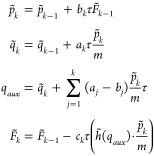
4where *m* is the mass and *p̃*_*k*_ = *p̃*(*t* = ∑_*j* = 1_^*k*^*b*_*j*_τ) and *q̃*_*k*_ = *q̃*(*t* = ∑ _*j* = 1_^*k*^*a*_*j*_τ) are the momentum and position (vector) variables at step *k* of the symplectic map starting from the initial conditions *p̃*_0_, *q̃*_0_, and *F̃*_0_ = *∂ V* (*q̃*_0_)/*∂q̃*. The numerical coefficients *a*_*k*_, *b*_*k*_, and *c*_*k*_ are universal real numbers that depend only on the order of approximation. Elegant derivations of the *a*_*k*_ and *b*_*k*_ coefficients for high order integrators can be found in the literature, as many authors have worked in the field of symplectic integration.^41−43^ Thus, in the next sections, we give only a brief overview for the derivation of the *a*_*k*_ and *b*_*k*_ coefficients, which are solutions of the system of equations given in the Appendix (we leave a more in depth explanation in the Supporting Information material). In the next sections, we discuss in some more detail how the *c*_*k*_ coefficients can be easily obtained for a second order integrator and how the fourth and higher order integrators can be obtained by composition of second order ones. For a second order integration, we found the unique solution *b*_1_ = 0, *b*_2_ = 1; *a*_1_ = *c*_1_ = 1/2, *a*_2_ = *c*_2_ = 1/2, which corresponds to the symplectic leapfrog algorithm with *c*_*k*_ = *a*_*k*_. We then propose a fourth order version of the pair-decoupled algorithm as a symmetric product of three leapfrog algorithms, with coefficients *a*_1_ = *a*_4_ = (2^1/3^ + 2^–1/3^ + 2)/6; *a*_2_ = *a*_3_ = −(2^1/3^ + 2^–1/3^ – 1)/6; *b*_1_ = 0; *b*_2_ = *b*_4_ = (2^4/3^ + 2^2/3^ + 4)/6; *b*_3_ = −(2^7/3^ + 2^5/3^ + 2)/6, *c*_*k*_ = *a*_*k*_. This choice is the most accurate, according to our numerical tests. However, several other choices arise when the higher order algorithms are not derived as symmetric products of lower order algorithms, as discussed in the next sections and more in detail in the Supporting Information of this paper.

### Overview of Hamiltonian Systems Integration with Symplectic Maps

To derive the *a*_*k*_, *b*_*k*_, and *c*_*k*_ coefficients, we begin with the formal solution of the equations of motion
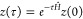
5where  is an operator that transforms the state function *z*(*t*) into its time derivative. In quantum mechanics, *z*(*t*) is a complex-valued wave function and , where *Ĥ* is the quantum Hamiltonian operator in atomic units. In classical mechanics, *z*(*t*) is a real-valued vector of the canonical coordinates and , where *H* is the classical Hamiltonian function and { *H*, ·} is called the Liouville (or Lie) operator, which works as a Poisson bracket of *z*(*t*):
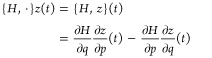
6We restrict our study to the dynamics of molecules, for which the nuclear part of the Hamiltonian can be written as a sum of a kinetic energy and a potential energy; that is, . Hence, the time evolution operator becomes . Since  and  do not commute, the exact time evolution operator is not just the product of its kinetic and potential components. A way to link  with products of kinetic and potential evolution operators^42^ is via the Baker-Campbell-Housdorff-Dynkin formula (BCHD), which is an infinite series of nested commutators and it can not be implemented directly. Nonetheless, the BCHD formula has been employed to demonstrate the time-invariance properties of even-order symplectic integrators.^41^ In case the Hamiltonian is not separable in its kinetic and potential contributions, it would still be possible to use the standard symplectic integration methods, by evolving copies of the system onto an extended phase space, as described by Tao.^44^

Two very common approximations for  are the first order map 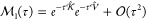
and the second order map 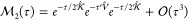
The latter approximation is known by various names, depending on the context (Strang splitting^45^ by mathematicians, Trotter-Suzuki splitting^46^ in the quantum mechanics community, Symplectic Leapfrog or explicit Verlet in the classical mechanics community).^47^ We will refer to the second order map as “SE2” (Symplectic Explicit of 2^*nd*^ order).

A more general approximation of the time evolution operator is given by
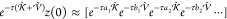
7with the constraints ∑_*k*_*a*_*k*_ = ∑_*k*_*b*_*k*_ = 1. Many approximate solutions in the *a*_*k*_ and *b*_*k*_ variables have been found in the ’80s and ’90s (assuming the classical expressions for  and ).^41,48−54^ A major advancement was done by Creutz,^55^ Yoshida,^41^ and Suzuki,^46^ who independently derived (among other things) a general formula for a class of arbitrary even order integrators using a “symmetric product”^41,44,46,50,55^ of lower order symplectic maps 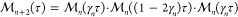
with . In particular, the symmetric product or SE2 algorithms, gives the well-known fourth order symplectic map with the coefficients reported by Forest and Ruth.^50^ Also notice that the symmetric product formula holds true even if we change the form of the  and  operators, meaning that we can apply it to our customized pair-decoupling Hamiltonian.

We also consider the coefficients presented in ref (56), which provide a very accurate fourth order integrator that, however, cannot be obtained as a symmetric product of second order integrators and it is not time-reversible by construction. In the Supporting Information of this paper, we report a general derivation of symplectic maps up to fourth order, from which we can derive both fourth order integrators along with their pair-decoupled versions. Our approach to derive the *a*_*k*_, *b*_*k*_, *c*_*k*_ coefficients consists of the direct application of the operators in eqs 7 or 4 to *z*(0) for a given truncation of the map (for a given integer *n*), followed by a comparison of the resulting *z*(τ) with the time Taylor series of *z*(*t*) centered in *z*(0) at the same orders of τ.

We point out, for sake of completeness, that there are also other ways of writing a symplectic map, for instance, explicitly including third order terms of the BCHD formula, such as , into the map in eq 7, where the commutator, assuming classical mechanics, evaluates to 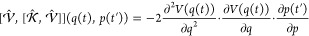
This approach was presented by Suzuki, in an attempt to derive an integrator with only positive *a*_*k*_ and *b*_*k*_ coefficients and avoid the unboundedness of the propagators with otherwise positive exponents (which make no sense when applied to diffusion algorithms).^57^ The actual integrator with positive-only coefficients was derived and implemented by Chin,^54^ and is proven to be extremely accurate, but also more expensive, because it requires evaluation of the Hessian matrix. While this method might be appropriate to integrate a pair-decoupling integrator, we disregarded it because of the difficulties of having the derivatives of the potential evaluated at the same time *t*, as should be clear from the next section of this paper.

The developments in this work can also be applied in different contexts, although they might require different interpretations. We collect here some of the recent developments in the use of geometric and symplectic integrators in the context of simulating quantum mechanical systems using classical trajectory methods, that could make use of the pair-decoupling integrators straightforwardly.^58−64^

### Integration of the Pair-Decoupled System

We construct our algorithm to be of the type of a *n*th order symplectic map
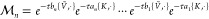
8which consists of a time evolution of a free system, followed by a time evolution of the pair-decoupled system with zero velocity, followed by evolution of the free system and so on. Notice that, in comparison with the standard symplectic map in eqs 7, we modify only the form of the potential energy operator , without changing the structure of the map, which remain symplectic, independently of how we modify . In fact, as long as eq 8 can be written as a single product of time evolution operators, we are sure that symplectic structure is preserved, contrary, for instance, to standard Runge-Kutta-Nystrom algorithms, which cannot be written as a single product, as explained by Chin.^43^ We use the definition given in eq 1 to integrate the Hessian and get the locally harmonic approximated expression for the (pair-decoupled) force
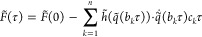
9The positions and momenta are those resulting from the application of the operators in the symplectic map of eq 8. Notice that in eq 9, one needs *q̃* and  to be evaluated at the same time, *t* = *b*_*k*_τ. However, after the application of the two rightmost operators of eq 8, one obtains the position and the velocity at different time values, i.e., *q̃*(*a*_1_τ) and , since, in general, *a*_1_ ≠ *b*_1_. Hence, we introduce an auxiliary position variable 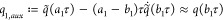
which is the position estimate at the same instant of time of the conjugated momentum variable. In general, 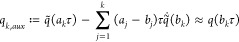
In this way, the integration of the force in eq 9 is consistent with the rest of the algorithm. Finally, given eq 9, we can compare the explicit form of the evolution operator for the pair-decoupled potential with the evolution operator of the original potential. To do that, we first evolve *z*(0) until we get (*p*(*t*_*p*_), *q*(*t*_*q*_)) = *e*^–*τ a*_*k*_{ *K*,·}^∏_*i*_^*k*–*τ b*_*i*_{ *Ṽ*,·}^*e*^–*τ a*_*i*_{ *K*,·}^)*z*(0), where we call *t*_*p*_ = ∑_*i*_^*k*^*a*_*i*_τ and *t*_*q*_ = ∑_*i*_^*k*–1^*b*_*i*_τ the time arguments of the momentum and position variables. Then, the additional application of the evolution operator *e*^–*τ b*_*k*_{ *Ṽ*,·}^ gives
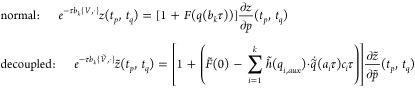
10We call , *p̃*_*k*_ ≔ *p̃*_*k*–1_ + *b*_*k*_*τF̃*_*k*–1_, and *q̃*_*k*_ ≔ *q̃*_*k*_ + *a*_*k*_*τp̃*_*k*_ the variables we need to store to implement the algorithm. We determine the coefficients *c*_*k*_ by considering that in the case of no decoupling (α = 1) the pair decoupled algorithm must provide a good estimate of the original force, i.e., *F̃*_*k*_ ≈ *F*_*k*_, and of the pair-decoupled position and momentum. Hence, a route to find the *c*_*k*_ coefficients is to assume that *a*_*k*_ and *b*_*k*_ coefficients are equal to those derived for the fully coupled system and then to choose the *c*_*k*_ coefficients so that the errors on *q̃*_*n*_ and *p̃*_*n*_ (and *F̃*_*n*_) are of a given order of τ when α = 1. Notice that, in the particular case of a quadratic potential, *h̃* in eq 10 is a constant (and *F̃*(0) = 0, assuming that at *t* = 0 the system is in equilibrium). Thus, for a quadratic potential, we have 
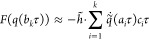
This is a good estimate of the force when the sum  is a good estimate of the position, that is, when *c*_*i*_ = *a*_*i*_. In fact, the appealing choice *c*_*k*_ = *a*_*k*_ is appropriate also for non-quadratic potentials when using a second order integrator (that is, either *a*_1_ = *a*_2_ = 1/2, *b*_1_ = 0, *b*_2_ = 1, or *b*_1_ = *b*_2_ = 1/2, *a*_1_ = 0, *a*_2_ = 1). This is not surprising, because the expanded expression for  contains the derivatives of *V* (*q*) and *K*(*p*) up to the second order, meaning that it is, in fact, a propagator of the system under a local quadratic approximation of the exact functions *K*(*p̃*), which is actually quadratic, and *Ṽ*(*q̃*), which is not. We call this choice the “SEF2” method (Symplectic Explicit with Force integration of the second order). The full set of coefficients is reported in the corresponding lines of Table 1.

**Table 1:** Summary of the *a*_*k*_, *b*_*k*_, and *c*_*k*_ Coefficients for Various Versions of the SEF Algorithm

SEF version	coefficients	*k* = 1	*k* = 2	*k* = 3	*k* = 4
SEF2^a^	*a*_*k*_	1/2	1/2	0	0
	*b*_*k*_	0	1	0	0
	*c*_*k*_	1/2	1/2	0	0
SEF4^b^	*a*_*k*_	(2^1/3^ + 2^–1/3^ + 2)/6	– (2^1/3^ + 2^–1/3^ – 1)/6	– (2^1/3^ + 2^–1/3^ – 1)/6	(2^1/3^ + 2^–1/3^ + 2)/6
	*b*_*k*_	0	(2^4/3^ + 2^2/3^ + 4)/6	– (2^7/3^ + 2^5/3^ + 2)/6	(2^4/3^ + 2^2/3^ + 4)/6
	*c*_*k*_	(2^1/3^ + 2^–1/3^ + 2)/6	– (2^1/3^ + 2^–1/3^ – 1)/6	– (2^1/3^ + 2^–1/3^ – 1)/6	(2^1/3^ + 2^–1/3^ + 2)/6
SEF4-I^c^	*a*_*k*_				+ 1/2
	*b*_*k*_	0		1/2	+ 1/4
	*c*_*k*_	5/26			5/26
SEF4-II^c^	*a*_*k*_	+ 1/2			+ 1/2
	*b*_*k*_	0		1/2	+ 1/4
	*c*_*k*_		+ 1/2		
SEF4-III^c^	*a*_*k*_				+ 1/2
	*b*_*k*_	0		1/2	+ 1/4
	*c*_*k*_	1/4			1/4
SEF4-IV^c^	*a*_*k*_				+ 1/2
	*b*_*k*_	0		1/2	+ 1/4
	*c*_*k*_		1/2	1/2	

a Using Symplectic Leapfrog *a*_*k*_ and *b*_*k*_coefficients.

b Using Forest and Ruth *a*_*k*_ and *b*_*k*_ coefficients.^50^

c Using Brewer, Hulme, and Manolopoulos *a*_*k*_ and *b*_*k*_ coefficients.^56^

The obvious way to derive an integrator of order four and higher is by composing lower order integrators, as mentioned above. The fourth order SEF4 integrator can be obtained as a symmetric product of the second order SEF2, with *a*_*k*_ and *b*_*k*_ coefficients equal to those derived by Forest and Ruth (also, independently derived by Campostrini and Rossi, and Candy and Rozmus),^49,53^ and with *c*_*k*_ = *a*_*k*_. All of the coefficients are reported in the SEF4 row of Table 1. Integrators of order 6 and higher can be easily obtained in the same way, resulting in the coefficients reported by Yoshida,^41^ with *c*_*k*_ = *a*_*k*_. However, the method of composing lower order integrators does not generate all the solutions to the fourth order symplectic map in eq 7. All such solutions can be found by solving the system in eq 19 of the Appendix, where, however, time reversible symmetry is not enforced. In particular, the coefficients reported in Appendix of ref are solutions to eq 16, but do not enforce time reversibility, despite providing an integrator that is more accurate than Forest and Ruth’s (in terms of energy conservation). While it is possible to build a pair-decoupled integrator using the *a*_*k*_ and *b*_*k*_ coefficients by Brewer et al., it is not possible to reach fourth order accuracy, and there are multiple possible choices of the *c*_*k*_ coefficients that we discuss in the Supporting Information. All such coefficients are reported in the rows SEF4-I to SEF4-IV of Table 1, as possible variants of the SEF4 integrator.

In the Numerical tests section we show numerically that our modification of the symplectic algorithm, which accounts for the pair-decoupling concept, preserves the properties of symplectic integration. Considering the Jacobian matrix
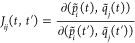
11and the canonical symplectic matrix
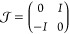
12we measure how much the relation  holds true for the special case *t′* = 0. In the case of a quadratic potential, the time evolution is exactly time reversible. Instead, in the case of a generic potential, the time-reversibility property is only approximate.

### Computational Details

In order to test our algorithm of eq 4, we employ accurate quartic force fields^65,66^ for the simulations of water and formaldehyde molecules. Instead, for salicylic acid calculations, we use the fitted potential energy DFT surface^67,68^ provided alongside the sGDML software.^67−73^ The sGDML PES is given already trained^67,68^ on 1000 training points, and it showed, with the inclusion of the Tkatchenko-Scheffler correction to account for the van der Waals interactions,^74^ a mean absolute error (MAE) which is less than 0.12 kcal/mol with respect to the pVDZ/DFT-PBE values.

To test the accuracy of the integration technique, we run 4 types of tests with α = 1. First we check the energy conservation along the simulation. Then, we checked the symplectic property of the Jacobian matrix. Then, we check the time-reversibility of the integrator, and eventually we compute also the classical power spectrum. The check of energy conservation may appear redundant because a symplectic integration, by definition, implies that all the constants of motion are preserved. However, our approximation of the force implies that the system evolved under an approximation of the potential. As a matter of fact, even if α = 1, if the force estimate in eq 9 is not accurate, the energy (of the fully coupled system) might not be accurately conserved, while the integration remains symplectic on the approximated potential.

To prove that the Jacobian matrix *J*(*t*, *t′*) is symplectic we use the relation  for the special case *t′* = 0, as anticipated above. The Jacobian with *t′* = 0 is called the monodromy matrix *M*(*t*), which can be computed numerically with the extended version of the algorithm described in the Supporting Information. Hence, we asses the stringent condition^12^
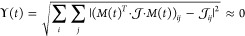
13Although eq 13 proves the symplectic property of the Jacobian matrix only for the special case *t′* = 0, this is the most stringent test from the numerical point of view.

To measure the degree of time reversibility of the integrator, we run a simulation until time *T*, with a 10 au time step. After that, we invert the sign of the momentum variable, and we continue the propagation for another time lapse equal to *T* backward, until a total simulation time of 2*T* is reached. Finally, we measure the quantity
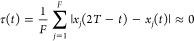
14where *F* = 3*N*_*at*_ (*N*_*at*_ is the number of atoms) and *x*_*j*_ is the *j*^*th*^ element of the *F*-dimensional Cartesian geometry vector. In all our tests, *T* = 6000 au.

Finally, we apply our integration technique for the calculation of the vibrational spectra, ranging from small molecules up to the salicylic acid molecule in the gas phase. We use a numerically convenient formula^75^ to evaluate the power spectrum of the *j*^*th*^ mass-scaled normal mode,
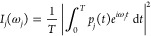
15This formula provides a resolved power spectrum with short (≈ 0.6 ps) simulations. In fact, eq 15 is the classical analogue of the time-averaging method employed in semiclassical spectroscopy.^76,77^ Since eq 15 computes a power spectrum from the velocity correlation function, all vibrational frequencies are reproduced and they can be compared with either infrared or Raman experimental frequencies. Instead, the intensities *I*(ω_*j*_) are not comparable with IR or Raman experiments because they depend only on the number of times the vibrational mode with frequency ω_*j*_ has occurred during the simulation time, which depends ultimately on the trajectory initial conditions. On the other hand, the experimental infrared and Raman intensities depend, for example, on the transition dipole moments and on the polarizabilities. To represent the power spectrum intensity of a multidimensional system we simply compute the sum of the power spectra of eq 15, that is
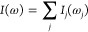
16The definite integral of eq 16 over a frequency domain can be interpreted as the average kinetic energy of the modes of vibration within that frequency domain.^33^ Thus, when the intensity *I*(ω) of the pair-decoupled simulation is different from the nondecoupled one, there must be a shift in the vibrational frequency or an intramolecular vibrational energy redistribution caused by the decoupling. The two effects may occur at the same time.

Although all the integrators described in this paper allow the system to evolve in any full-dimensional coordinate system, we always employ mass-scaled normal modes, which have the advantage of discarding translational and rotational motion. To decouple the Cartesian degrees of freedom, we just rotate the normal mode Hessian matrix to Cartesian coordinates, apply the decoupling, and rotate the decoupled matrix back to normal modes. We use this procedure, instead of evolving in Cartesian coordinates, because the SEF algorithm cannot accurately describe free translations and rotations or other types of motion that have a very flat (in general very anharmonic) potential landscape.

All of the simulations described in this paper are full dimensional and start from the equilibrium geometry of the fully coupled system. The normal mode coordinates are constructed using the fully coupled Hessian matrix. Also, in case the SEF algorithm is used, the initial force is assumed to be 0, just as if the initial geometry were an energy minimum for the pair-decoupled system, as well. The initial momentum in normal mode coordinates is set equal to the square root of the corresponding harmonic frequency so that the initial kinetic energy is equal to the harmonic zero point energy. In the simulations with α ≠ 1 we follow the same recipe, but, when the effect of the decoupling is weak, we run the simulations for longer time (5000 time steps), and discard the initial 2000 steps (which is about 0.5 ps), to allow the decoupled fragments to actually decorrelate. In some cases, such as when decoupling all the functional groups of salicylic acid, the decoupling effect is very strong, and the decorrelation effects can be seen already from the very beginning of the simulation. In such cases, we run the simulation for only 3000 steps and discard none. Anyway, if the pairs of atoms are naturally independent, the normal spectrum and the pair-decoupled spectrum would be exactly the same.

## Results

### Numerical Tests

**Figure 2:**
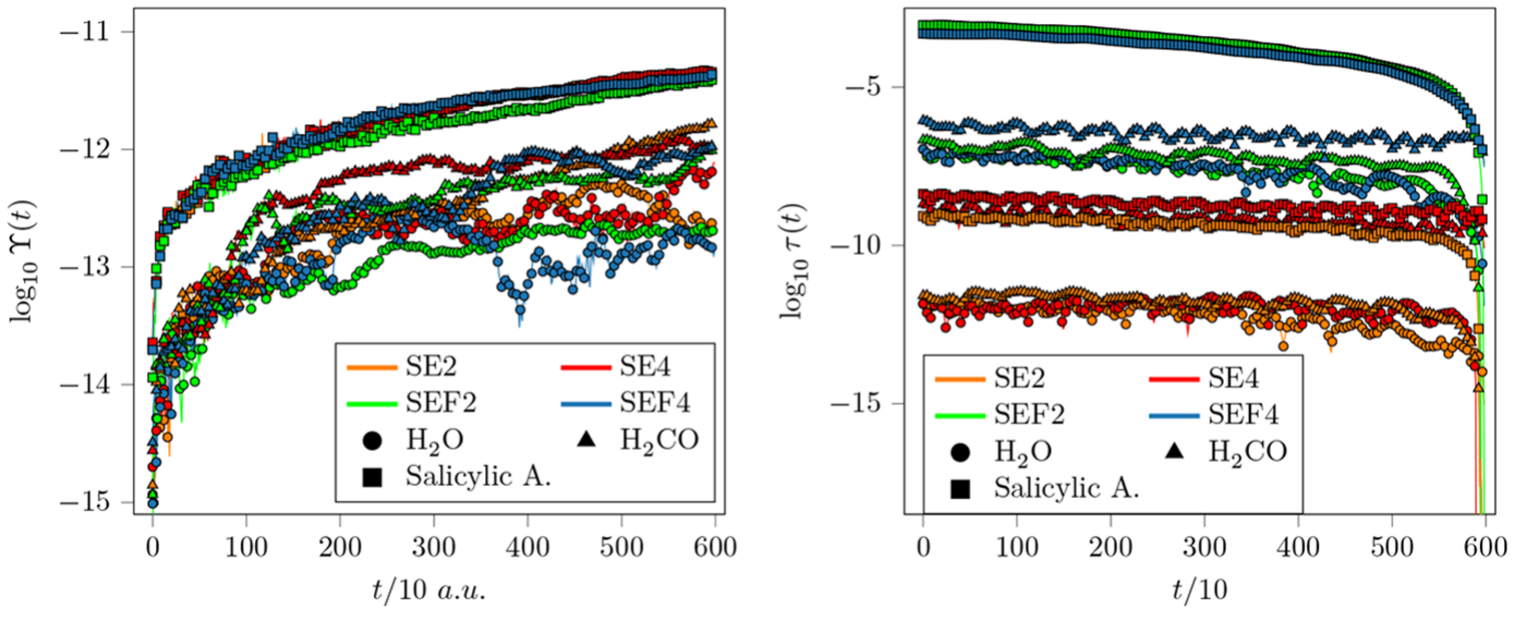
Υ(*t*) and τ(*t*) for a 3000 time-step simulation of 10 au each. SE2 (orange), SE4 (red), SEF2 (green), and SEF4 (blue) integration methods for H_2_O (circles), H_2_CO (triangles), and salicylic acid (squares). SEF2 and SEF4 are tested without decoupling.

We start by testing the accuracy of our algorithms. In Figure 2 we show how much the SEF2 and SEF4 integrators with α = 1 preserve the symplectic symmetry of the monodromy matrix *M*(*t*) and the time reversibility property. These are compared with the well established Symplectic Leapfrog (SE2) and fourth order SE4 method, that is the Symplectic Explicit integration method with the coefficients of Forest and Ruth.^50^ Independently of the integrator, the larger is the system, the quicker Υ(*t*) and τ(*t*) deteriorate, and this is mainly due to the fact that more operations are carried out in a finite precision arithmetic. However, when switching from the SE to the SEF algorithms, no significant further errors in Υ(*t*) are introduced, while the time reversibility accuracy is decreased by orders of magnitude. This is expected for two main reasons. First, the calculation of the force in the SEF algorithms is performed by time integration and it requires four sums of matrix multiplications. The second, and the most important one, is that the calculation of the force is based on a local harmonic approximation of the potential landscape. Our approximate evolution of the force within the local harmonic approximation is not a time-reversible process, except for quadratic potentials. These limitations are clearly amplified with the dimensionality.

The symplectic properties of the SEF integration are preserved also in the case of the decoupled pairs of degrees of freedom, i.e., α = 0. We show in the Supporting Information (Figure S1) that Υ(*t*) and τ(*t*) have the same shape even for the pair-decoupled system.

**Figure 3:**
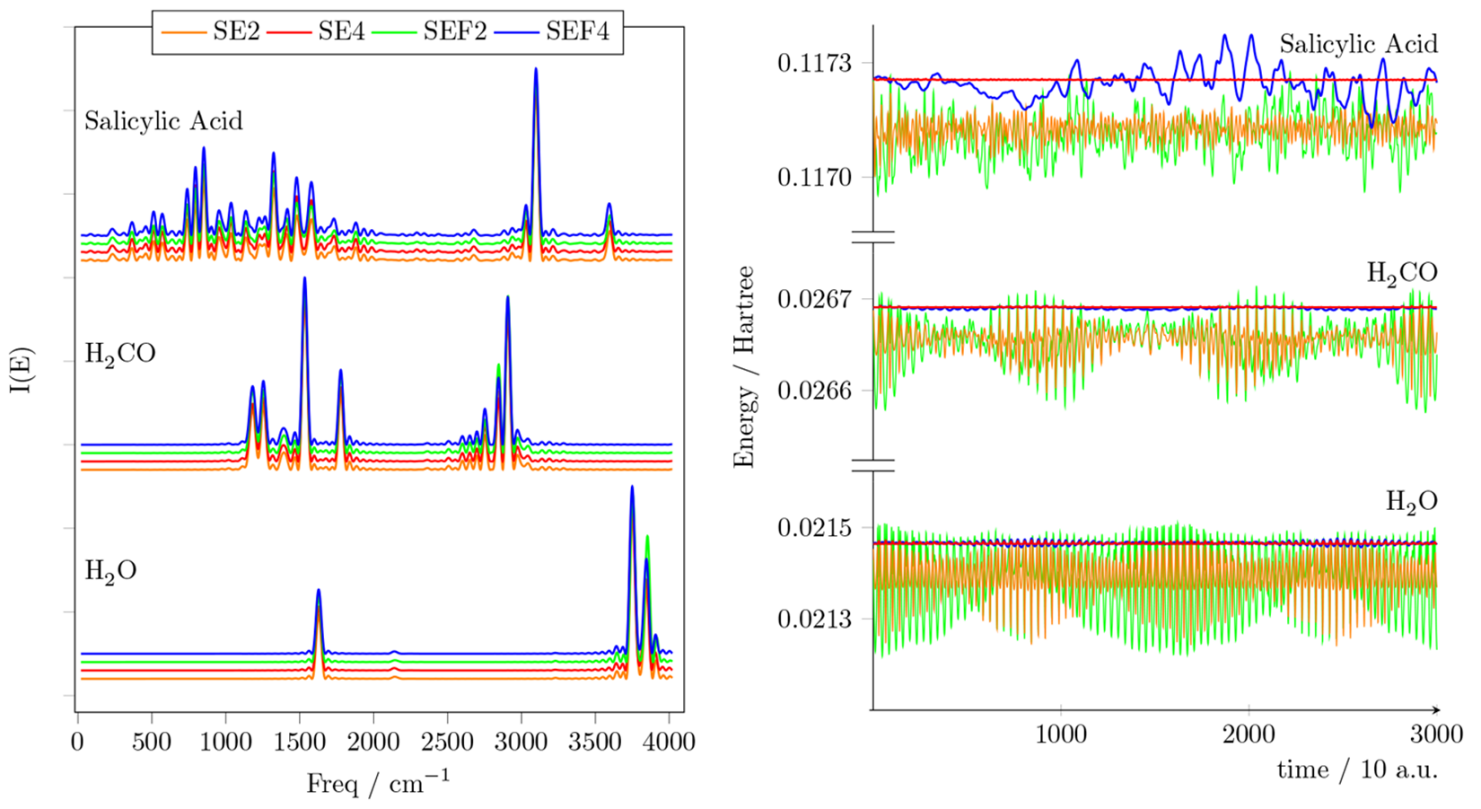
Power spectra and energy conservation. The left panel shows the power spectra of H_2_O, H_2_CO, and salicylic acid molecules computed with SE2 (orange), SE4 (red), SEF2 (green), and SEF4 (blue) integrators (always with α = 1). The right panel shows the energy profiles of the corresponding simulations.

In Figure 3 we show that, for all systems, the spectroscopic features are perfectly captured by the entire integration method. SEF4, SE2, and SE4 provide spectra that are almost quantitatively equivalent when applied to all systems. The total energy of the H_2_O and H_2_CO systems is well conserved by the SEF algorithms, with SEF2 having an oscillation that is about ∼10% larger than SE2, and SEF4 having an oscillation that is ∼20% to ∼30% larger than SE4. When the system includes floppy modes, however, such as the salicylic acid, these modes induce a slow oscillatory pattern in the energy profile that is not well captured by the SEF algorithm. In fact, SEF can not predict very accurately the strongly anharmonic contributions to the force. Nevertheless, this is not an issue, because the SEF energy does not display a systematic drift, but only a slow oscillatory pattern that follows the oscillation of the low energy modes.

### Decoupling the Salicylic Acid Fragments

Since the pair decoupling is, by definition, an artificial procedure, we rationalize the following results in a *reductio ad absurdum* style, where first we enforce that some fragments of the molecule are independent and simulate the corresponding system, and then we see how much the vibrational features are affected by the decoupling. In this way, we can observe that decoupling some fragments of the salicylic acid does not lead to significant conformational changes within a short simulation time, while decoupling other fragments quickly leads to unrealistic phenomena. However, given a long enough simulation time, decoupling any pairs of molecular fragments will eventually lead to unphysical behaviors.

**Figure 4:**
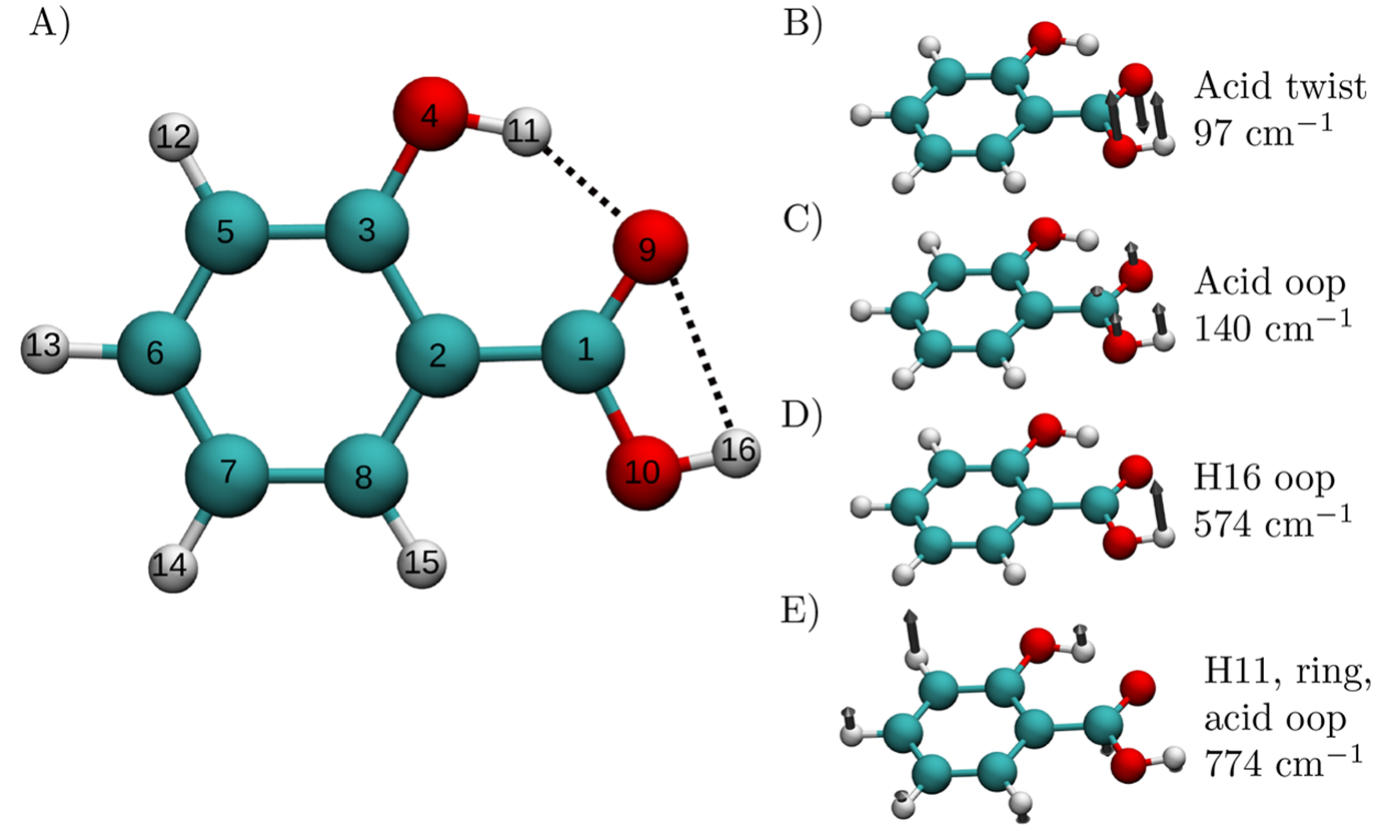
(A) Picture of salicylic acid with labeled atoms and (B, C, D, E) some relevant types of motion^83^ involving the acid group, H11, and H16 with their relative harmonic frequencies.

Previous infrared spectroscopic studies of salicylic acid (SA) focused primarily on the intramolecular H-bond between hydrogen 11 and oxygen 9^78−82^ and hydrogen 11 and oxygen 10^78^ (see the atom numbering in Figure 4) in the ground and first excited electronic states. These studies are mainly about the proton transfer process and deactivation of the excited electronic state via a radiationless mechanism, which is possible only if O9 and H11 are close enough, as shown in panel A of Figure 4, which is at the equilibrium geometry of the ground state. Below we show that such a configurational arrangement is stable over time only if the motion of O9 and H11, as well as of O9 and H16, is correlated. Furthermore, we investigate how much the carboxyl O – H stretching motion changes after artificially decoupling the different functional groups of the molecule.

#### Modes of Vibration of the Salicylic Acid

The salicylic acid molecule in its minimum energy geometry and within the harmonic approximation has 42 normal modes of vibration. Only some of them show a significant displacement of H11 or H16. However, beyond this approximation, the vibration of the hydroxyl and carboxylic acid fragments implies the significant displacement of H11 and H16 from their equilibrium condition. More specifically, approximating the O4–H11 and O10–H16 stretching modes with the harmonic approximation implies neglecting the coupling between these stretching modes and the twist, wag, and other complex motions that involve the whole OH and acid fragments. Four low frequency normal modes which are crucial for the salicylic acid vibrational motion are reported in panels B, C, and D of Figure 4. These modes are the acid group twist and the acid group out-of-plane (oop) modes, which involve, respectively, a twist and oop wagging of the carboxyl group with respect to the ring and the H16 oop motion, which is an out of plane wag of the H16 hydrogen. In addition, panel E of Figure 4 represents an out-of-plane mode that is delocalized over the three functional groups, involving H11, the ring, and the carboxylic acid group. We find that these four types of motion are those that are most significantly influenced by the pair decoupling of the hydrogen-bonded fragments and of the functional groups of SA. There are two main reasons for this. One is that they involve flexible regions of the molecule that easily couple with many other types of motion, and the other is that they break the directionality of the intramolecular hydrogen bonds.

An estimate of the vibrational frequencies of the pair decoupled system cannot be straightforwardly made within the harmonic approximation. In fact, while scaling the off-diagonal entries of the Hessian does not change the trace, which is conserved in the diagonalization, it might change the magnitude of the eigenvalues. These changes imply that the pair-decoupled Hessian does not correspond to a stationary point configuration anymore. Consider, for instance, the case when the carboxyl group is decoupled from the hydroxyl groups; that is, all of the atoms in the carboxyl group are fully decoupled from the O and H atoms in the hydroxyl group. The normal-mode analysis of such a system at the original equilibrium geometry shows that the now unhindered acid twist mode has a frequency of about 3650 cm^–1^. This is clearly not realistic. As mentioned above, the reason a normal-mode analysis can not be employed for an artificially decoupling analysis is that the equilibrium geometry of the system, at which the Hessian matrix is computed, is not a stationary point for the pair-decoupled system. Instead, computing the vibrational spectrum from the velocity correlation function does not suffer from this problem, and it can account for both anharmonicities, nonequilibrium, and dynamical couplings, which are lacking in the harmonic approximation.

#### Decoupling the O10–H16 and C1=O9 Stretching Modes

**Figure 5:**
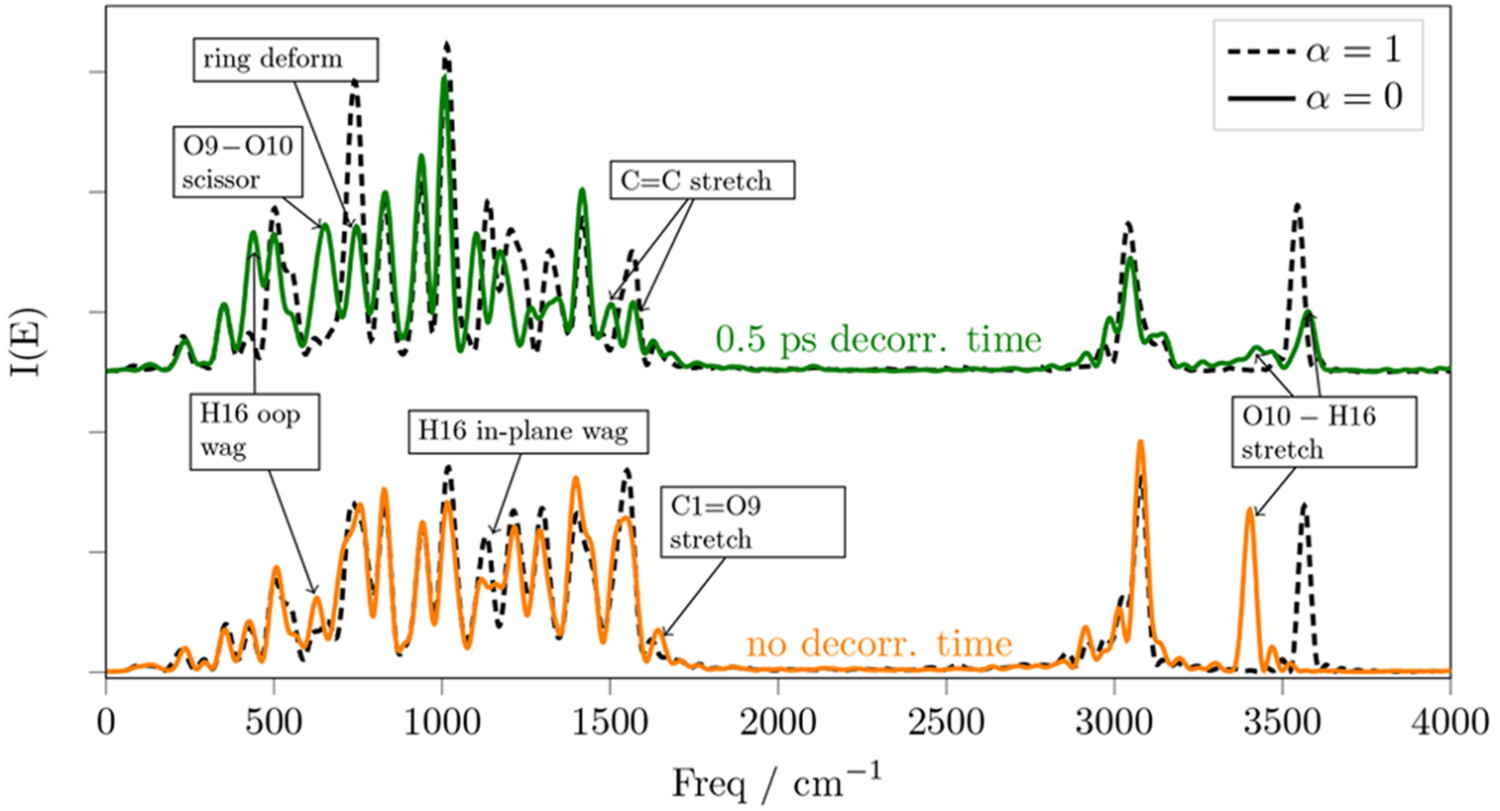
Power spectra of the salicylic acid (black dashed lines) and of the decoupled (α = 0) C1–O10 and O10–H16 stretching modes, without waiting for any decorrelation time (solid orange line) and after 0.5 ps of decorrelation time (solid green line). The α = 1 spectra are taken over the same time intervals of the corresponding α = 0 spectra, accounting for the decorrelation time.

In this section, we apply the pair-decoupling idea in normal mode coordinates. In particular, we decorrelate the O–H stretching mode from the C=O stretching mode of the SA. Both the O–H and C=O stretchings are localized, meaning that we can interpret the O–H and C=O as two oscillators, where the O and H, and C and O, atoms are each connected by a spring. Even though the two oscillators are defined as independent when the molecule is at equilibrium, outside of equilibrium, the two oscillators are coupled, and each one depends on the other one’s displacement. Furthermore, both oscillators also couple with all of the other oscillators that compose the SA vibrations. All the observations that we can make about the spectra in Figure 5 originate from the in-plane oscillations localized on the carboxylic acid group.

In Figure 5 we see that if we do not wait for any decorrelation time, the anharmonic vibrational frequency of the O–H stretch is red-shifted by nearly 160 cm^–1^. This effect is evidently localized on the O–H, because the rest of the spectrum is only slightly changed by the decoupling. However, after 0.5 ps of decorrelation time, the decoupling also affects the O–C–O scissoring mode, as well as modes that involve deformations and C=C stretchings. Moreover, after 0.5 ps of decorrelation time, the O–H stretching frequency becomes again similar to that of the nondecoupled system. Let us first focus on the bottom part of Figure 5. O9 and H16 are connected by a nondirectional hydrogen bond, that is weakened when the C=O and O–H bonds are stretched, because the two stretchings move the two atoms further apart. As the two oscillators stretch, they do not retain their reciprocal phase, because of the difference in mass between O and H. However, when we apply the decoupling, atom H16, would keep feeling the effect of a nonstretched C1–O9 oscillator, while oscillating back and forth. Thus we can see that the O–H stretching is hampered by the stable O9–H16 hydrogen bond. And we can quantify the importance of this effect by measuring the red-shift, which amounts to about 160 cm^–1^.

Let us now focus on the top part of Figure 5. It shows that after 0.5 ps of decorrelation time, also the normal modes localized on the ring are affected by the decoupling. The involvement of the other modes implies a structural deformation of the entire molecule, compared to the nondecoupled dynamics. This brings the carboxylic acid O–H stretching frequency to about 3570 cm^–1^, which is slightly blue-shifted from the nondecoupled spectrum (dashed line). This effect cannot be explained with simple arguments, because it evidently involves the whole molecule. As a matter of fact, the only portions of the spectrum that appear almost unaffected by the decoupling are C–H and hydroxyl O–H stretching modes, as well as the 700 and 1050 cm^–1^ region, which involves some ring deformation and breathing modes.

#### Decoupling the Carboxyl O9–H16 Hydrogen Bond

**Figure 6:**
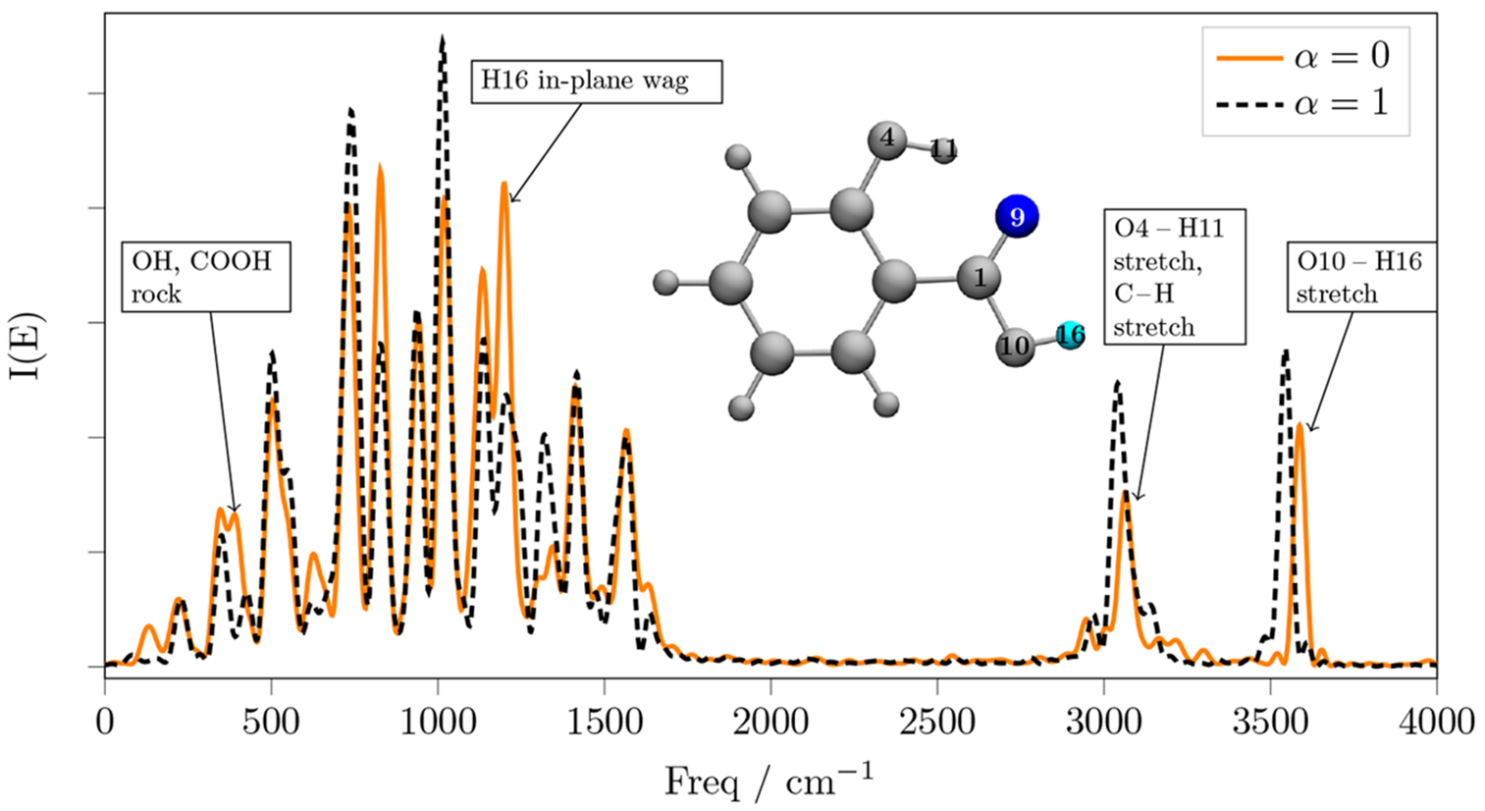
Power spectra of the salicylic acid (dashed black line) and of the O9–H16 decoupled (α = 0) salicylic acid (solid orange line). Both spectra are recorded after discarding the first 0.5 ps of simulation.

The first physical insight provided by the artificially decoupled O9–H16 hydrogen bond is that the O9 and H16 atoms do not oscillate synchronously anymore. This asynchronous motion induces an angular momentum that enhances the acid twist mode to the point that, after less than 300 fs, the carboxyl group attempts a 180° rotation around the C1–C2 axis. This 180 deg rotation has a potential barrier (computed as energy of the transition state minus energy of the minimum) in the original fully coupled system of 6366 cm^–1^ at pVDZ/DFT-PBE level of theory, and it should be an extremely rare event for the fully coupled system, considering that the acid twist motion is initialized with less than 100 cm^–1^ of kinetic energy. To avoid this artificial twist, which is not in a fitted region of the given sGDML potential energy surface, we run simulations where the acid twist normal mode is kept at equilibrium and the O9–H16 hydrogen bond is still decoupled. The power spectrum of this simulation after a 0.5 ps decorrelation time is shown in Figure 6. In this case, we can observe a rather weak decorrelation effect in terms of the enhanced H16 rock and in-plane wag. The effects of such enhanced motion are given by the more intense bands in the 400 to 500 cm^–1^ and 1100 to 1300 cm^–1^ regions, as well as by the blueshift of the O10–H16 stretching mode, indicating a slightly weaker bond between O10 and H16.

From a fixed nuclei picture of the SA in the minimum energy configuration, one assumes that the hydrogen bond between O9 and H16 ensures that the carboxyl group remains confined in a plane and that H16 is oriented toward O9. In a dynamical picture instead, H16 oscillates out of the O9–C1–O10 plane and, given enough energy, it might overcome the potential barrier and get oriented toward C8 in a 180° rotation around the C1–O10 axis. In the original fully coupled system, this barrier height is about 4355 cm^–1^ at the pVDZ/DFT-PBE level of theory, and thus, the 180° rotation around the C1–O10 axis is a rare event, considering that the H16 oop wagging is initialized with 574 cm^–1^ of kinetic energy. The lack of synchronization in the out-of-plane motion of O9 and H16 redistributes some of the stretching vibrational energy to the out-of-plane modes to the point that the rotations become allowed. In fact, if one keeps the carboxyl twist mode at equilibrium, the H16 oop wag begins to oscillate significantly after about 1.2 ps of simulation, and we observe an attempt of 180° rotation of O10–H16 around the C1–O10 axis.

To sum up, the simulations of the O9–H16 decoupled SA provide two main physical insights. First of all, the artificial decoupling allows one to appreciate the importance of the synchronous oop vibration without which the carboxyl group would rotate, leading to a less stable minimum configuration. Second, the asynchronous motion of O9 and H16 leads to a fast vibrational energy redistribution in favor of the out of plane modes. As a secondary result, we see that the pair decoupling allows one to quickly explore otherwise almost forbidden configurational regions of the potential surface.

#### Decoupling the Hydroxyl and Carboxylic Acid Groups

**Figure 7:**
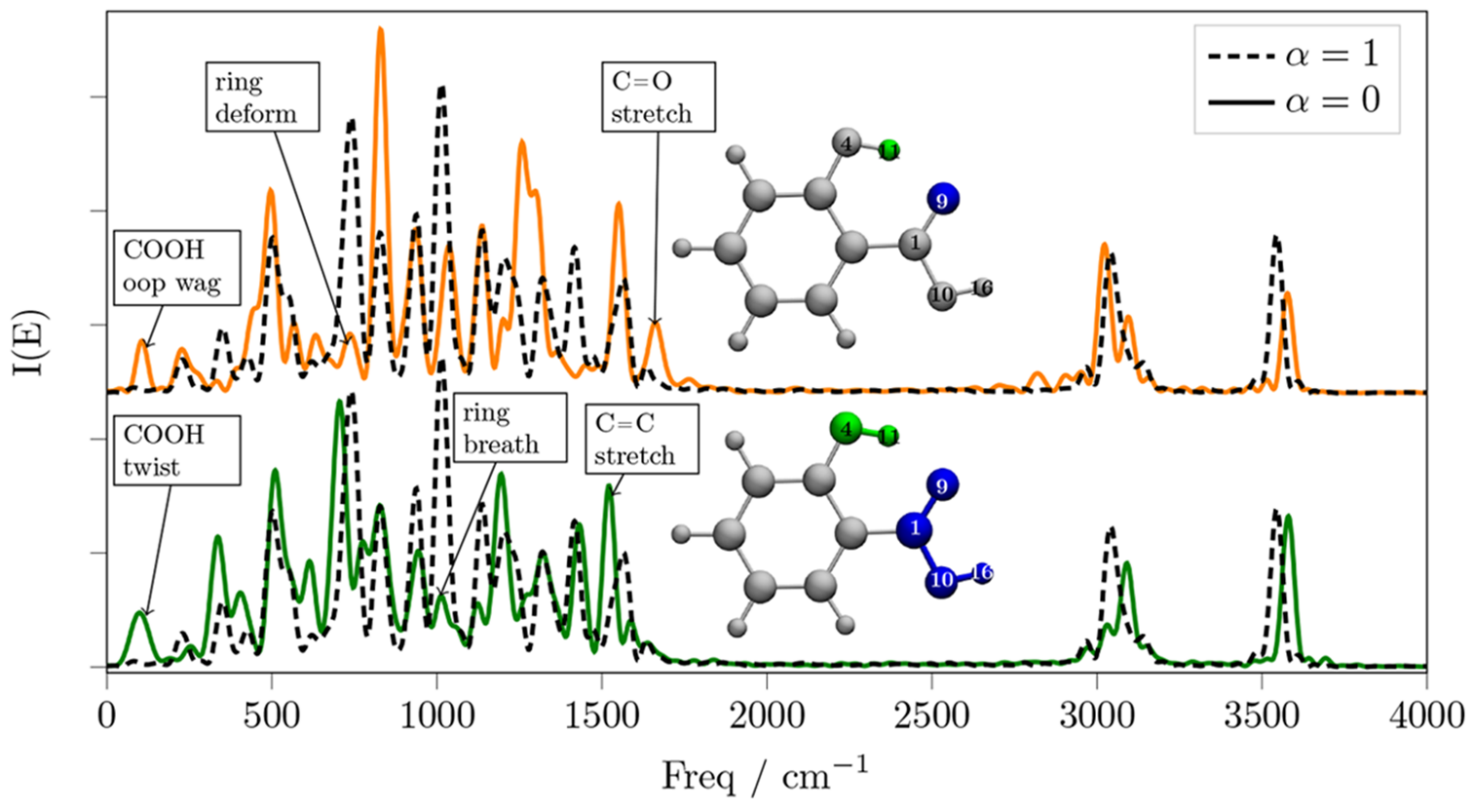
Power spectrum of the O9–H11 (solid orange line) and carboxyl–hydroxyl (solid green line) decoupled SA. The black dashed line is the power spectrum of the fully coupled simulation for comparison. The O9–H11 simulation (orange line) was obtained without evolving the acid twist mode, and both spectra are recorded after a 0.5 ps decorrelation time.

Here we show with our simulations why the O9–H11 hydrogen bond is fundamental for the planar shape of SA. As a consequence of the O9–H11 decoupling, the carboxyl group quickly initiates a large amplitude twist around the C1–C2 axis. This effect is similar to that one we have described in the previous section for the O9–H11 decoupling, where the decoupling artificially augments the kinetic energy of the acid twist motion represented in panel B of Figure 4. However, in this case, the role of the H-bond is very different. When O9 and H11 are decoupled, we argue that, even if the out-of-plane motions are not synchronized anymore, the oop wag of H11 remains coupled to O10 (and H16) and this coupling stimulates the oop motion of O10 (given the planarity of the carboxyl group). Eventually, the artificially enhanced oop wagging of H11 induces an attempted 180° rotation of the carboxyl group around the C1–C2 axis. We deduce that there must be a strong synchronized interaction of each atom composing the *whole* hydroxyl group with each atom composing the *whole* carboxyl group. This interpretation of the importance of the H11 interaction with each singular atom composing the carboxyl group is validated by the fact that the acid twist motion is not enhanced when the whole carboxyl group is decoupled from the entire hydroxyl group. In Figure 7 we show both the spectrum when the O9–H11 interaction is decoupled and the acid twist mode is kept at equilibrium, and also the spectrum when it is the carboxyl–hydroxyl entire groups to be decoupled. Both spectra are recorded after 0.5 ps of decorrelation time, and both spectra show that the decoupling effect is quite significant in terms of vibrational energy redistribution. In fact, the ring breathing and ring deformation modes donate vibrational energy to the out-of-plane and C=O stretching modes at about 100 to 400, and 1600 cm^–1^, respectively. More specifically, both the O9–H11 (orange line) and the carboxyl–hydroxyl decoupled spectra (green line) show that the decouplings induce significant vibrational energy redistributions in both the low frequency and fingerprint regions of the spectra, especially from the ring breathing and ring deformation modes, while the ring C–H and O–H stretching signals retain their kinetic energy on average. In both the O9–H11 and carboxyl–hydroxyl decouplings, the O–H stretching signals are mildly blue-shifted, indicating slightly weaker hydrogen bonds. In conclusion, these results clearly show that the intuitive picture of the independent functional groups in ortho position on the aromatic ring is partial to describing the appropriate vibrational dynamics of the SA and that the single atom–atom instantaneous couplings are essential for an accurate description of the interactions between the two functional groups. Our results also show that, surprisingly, the hydroxyl–carboxyl decoupled system provides a more realistic simulation of the O9–H11 decoupled one, because it does not induce the acid twist rotation.

#### Decoupling the Carboxyl and Ring Fragments

**Figure 8:**
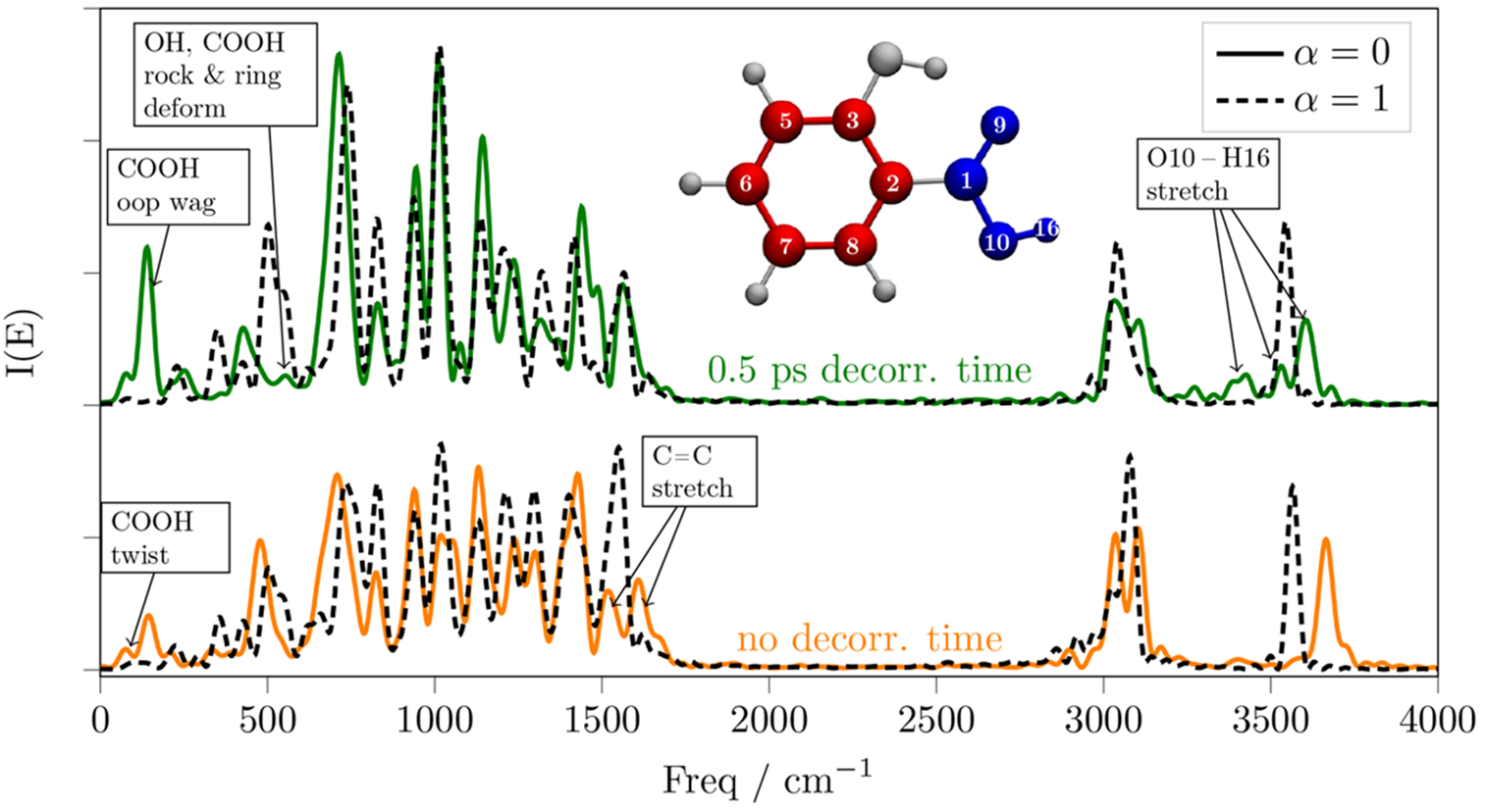
Spectrum of the SA with the carbon ring decoupled from the carboxyl group with α = 0, without waiting for any decorrelation time (orange line) and after 0.5 ps of decorrelation time (green line). The dashed black lines are the spectra of the fully coupled system after discarding the corresponding amount of simulation time to match the decorrelation time.

We find that the strongest decorrelation effects that impact the carboxyl O–H stretching mode occur when we decouple the motion of the ring from that of the carboxylic acid group. Specifically, the stretching O–H mode of the carboxylic acid is blueshifted by 103 cm^–1^ (from 3573 cm^–1^ of the fully coupled system to 3675 cm^–1^ of the α = 0 decoupled system), as shown by the solid orange line in Figure 8. This effect is accompanied by an increased amplitude H16 oop wagging motion, which shortly after about 0.7 ps induces a 180° rotation of the O–H around the C1–O10 axis. If we keep the H16 oop wag at its equilibrium geometry to avoid this artificial rotation and record the power spectrum after 0.5 ps of decorrelation time, the O–H stretching of the acid is still blueshifted, although only by about 50 cm^–1^, as shown by the solid green line of Figure 8. We conclude that the ring and carboxyl group decoupling have a strong effect on the carboxyl O–H motion. In fact, the C–H and hydroxyl O–H stretching signals around 3100 cm^–1^ are split, although not one of those hydrogens is decoupled.

The ring-carboxyl decoupling is a good example of how the two fragments can be interpreted as independently vibrating fragments just after the decoupling effect is turned on but not after 0.5 ps of decorrelation time. As a matter of fact, after the decorrelation time, the carboxyl O–H stretching signal is smeared over nearly 500 cm^–1^, mainly because of the enhanced oop wagging motion of the carboxyl group at 150 cm^–1^. Nonetheless, most of the signals that involve ring stretching and other motions delocalized over the ring are still well recognizable in the fingerprint region of the spectrum, even after the decorrelation time. This observation shows that the effects of substituents on the vibrational features of the ring are mostly static, i.e., decorrelating the ring and carboxyl group vibrations does not induce very significant changes in the ring vibration frequencies. In fact, the fingerprint region of the spectra, between 700 and 1500 cm^–1^, displays only some mild vibrational energy redistribution, in favors of the COOH twist and oop wag.

### Decoupling the Entire SA into a Ring Part and Its Substituents

We conclude the results section by describing the scenario in which the SA molecule is decomposed into a ring and its substituents.

**Figure 9:**
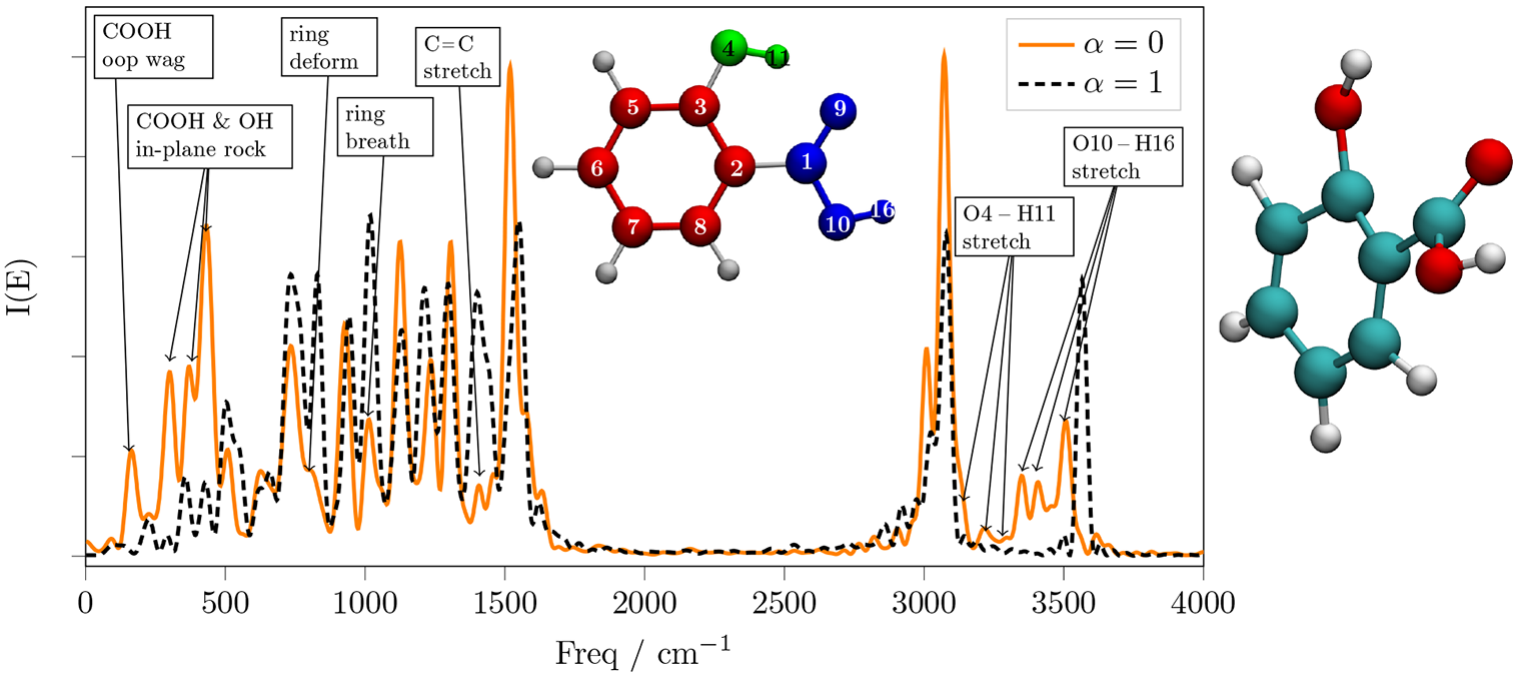
Spectrum of the SA with the carbon ring, carboxyl, and hydroxyl fragments all decoupled from each other with α = 0 (orange line). The dashed black line is the spectrum of the fully coupled system for comparison. The inset on the right shows a representative frame of the simulation, as a result of the asynchronous motion of the three decoupled fragments.

Figure 9 shows in orange the power spectrum of the SA where its functional groups are artificially decoupled. These groups are the aromatic carbon ring, the hydroxyl group, and the carboxyl group. After only 180 fs the decoupled system attempts a rotation of the O4–H11 group around the C3–O4 axis. Therefore, in this case, we decided to keep at the equilibrium position the mode which involves simultaneously the H11, ring, and acid oop displacements, which is indicated in panel E of Figure 4. Then, we record the spectrum without waiting for any decorrelation time. The strongest decorrelation effects observed in this dynamics consists of the enhanced acid twist, acid oop wag, and O–H oop wags, represented in panels B, C, and D of Figure 4. All of these effects quickly bring the molecule into very energetic regions of the PES, such as shown in the inset of Figure 9. From Figure 9 we can also see the same smearing of the carboxyl O–H stretching signal observed in the green spectrum of Figure 8, which occurs without any decorrelation time. This smearing effect is mainly due to the fact that the acid twists to a staggered position, and simultaneously, it also bends toward the ring (see the inset of Figure 9). In such a distorted configuration, the carboxyl O–H stretching motion strongly depends on the orientation of the O–H, as well as on the amount of twisting. In addition, the hydroxyl group is anomalously stretched apart from the ring and the C1–O10–H16 angle is highly increased, and similarly the signals of the hydroxyl O–H stretchings in the 3100 to 3300 cm^–1^ frequency interval are smeared. On the contrary the C–H stretching signals at about 3000 and 3100 cm^–1^ are only slightly shifted but not smeared. The redshift of the carboxyl stretching can also be interpreted from the distorted geometry of the SA. When the carboxyl group is twisted, its electron withdrawing effect on the ring is weakened and it becomes a weaker acid.

Analogously to what we observed in the ring–carboxyl decoupling of Figure 8, the fingerprint region of the spectrum remains reasonably similar (in terms of frequencies) to that of the fully coupled system. However, some of the ring modes, in particular the C=C stretchings, ring deformation, and breathing modes, donate vibrational energy to the hydroxyl and carboxyl groups in the 100 to 500 cm^–1^ region of the spectrum.

## Discussion and Conclusions

In this paper, we introduce a pair-decoupling idea that offers a novel perspective for the study of relationships among groups of atoms or, more generally, of degrees of freedom in a molecule. The pair-decoupling idea is based on a simple, yet always applicable, mathematical definition: the Hessian matrix of a pair decoupled system is equal to the Hessian matrix of a normal system where some of the off-diagonal elements are weighted with an arbitrary coefficient α. The SEF algorithm that we introduce enforces the pair-decoupling idea for the molecular dynamics simulations of small and medium sized organic molecules. The simulations faithfully preserve the properties of symplectic symmetry of classical dynamics, in particular, the phase space conservation, in agreement with Liouville’s theorem. The SEF method is effectively a symplectic integration technique of a system under a locally harmonic, “pair-decoupled” potential. The main disadvantage of the method is the requirement of 2 or 4 Hessian matrix calculations per time-step. However, this limitation could be alleviated by suitable numerical techniques.^84,85^ This unavoidable feature limits the employment to middle-sized molecules and imposes the use of a computationally affordable potential for the electronic structure.

The application of our technique to salicylic acid has shown both intuitive behaviors of the pair-decoupled system, such as the rotation of the carbonyl in response to a decoupling of the hydroxyl-carboxyl H-bond, and less intuitive and surprising effects, such as the blueshift of the carboxyl O–H stretching frequency when the acid hydrogen is decoupled from the aromatic ring. We also showed that the synchronous vibrations of the atoms in the carboxyl and hydroxyl fragments are essential for the equilibrium configuration to be stable over time. As a consequence, in the absence of such couplings, the proton transfer photochemistry of the salicylic acid would be impossible. Ultimately, our simulations of the pair-decoupled salicylic acid show that the picture of the molecule as composed of independent vibrating fragments is partial and often unreliable. Since this intuitive picture is at the origin of the functional groups definition, we think that these results show how there are important exceptions to the functional group picture. In fact, an artificial decoupling of apparently unrelated groups of atoms may induce evident changes in the vibrational spectroscopy of the whole molecule. We think that these considerations are applicable to many other chemical systems and that our results open the path to further investigations thanks to the computational tool that we have presented. Furthermore, the pair-decoupled simulation technique can be used to validate applications that assume that a portion of the system is partially uncoupled from another such as in MCTDH and QM/MM calculations. The most practical way to do that in the case of QM/MM, for example, is to simulate the chosen pair-decoupled system at the MM level and verify whether it is an appropriate partition for the QM/MM calculation.

We hope that the pair-decoupling idea can inspire other less computationally expensive methods that can assess the importance of couplings in molecules. Finally, we believe that the SEF algorithm can help increase the sensibility of chemists toward the (unexpected) effects of approximations that involve artificially decoupled systems.

## Data Availability

Data and computer codes are available upon reasonable request to the corresponding author.

## Appendix

The solutions of the fourth order map  are obtained from the following system of equations, which we derived, as explained in the Supporting Information, with the help of the SageMath^86^ computer algebra system:

17

It is important to notice that this system is undetermined. We need to choose another constraint to effectively get numerical values for *a*_*k*_ and *b*_*k*_ coefficients. A reasonable choice to saturate the system is to set *b*_1_ = 0, which leads to three possible sets of real coefficients: the first set was first published by Campostrini and Rossi,^49^ then independently by Forest and Ruth,^50^ Yoshida (who attributes the origin to Neri),^41^ and Candy and Rozmus.^53^

18

while the second was reported by Brewer et al.,^56^

19

## Supporting Information Available

A more detailed description of the strategy we used to derive the system in eq 16 of the Appendix; a more detailed description of the modified symplectic map, and of the choices of the *c*_*k*_ coefficients for SEF4-I to SEF4-IV variants; equations to implement the monodromy matrix time evolution alongside the SEF algorithm; pictures that show the Υ(*t*), τ(*t*) functions for the decoupled (α = 0) water molecule (PDF)
