# Supplementary material for: Molecular Dynamics of Artificially Pair-Decoupled Systems: An Accurate Tool for Investigating the Importance of Intramolecular Couplings
Source: J Chem Theory Comput. 2023 Sep 12;19(18):6093–108. doi: 10.1021/acs.jctc.3c00553 (PMC10536992; doi:10.1021/acs.jctc.3c00553)
Supplement: Supplementary file 1 — ct3c00553_si_001.pdf [file ct3c00553_si_001.pdf]

Molecular Dynamics of Artificially Pair-Decoupled Systems: An accurate tool for investigating the importance of intramolecular couplings

Michele Gandolfi and Michele Ceotto[1, a)]

Dipartimento di Chimica, Università degli Studi di Milano, via Golgi 19, 20133 Milano, Italy

a)Electronic mail: michele.ceotto@unimi.it

CONTENTS

I. Supporting Information S2
A. Details about the symplectic integration algorithms S2
B. Details about the decoupling symplectic integration algorithm S6
C. Evolution of the pair-decoupled Monodromy matrix S11

References S14

I. SUPPORTING INFORMATION

A. Details about the symplectic integration algorithms

Consider a classical Hamiltonian of the general type:

$$H = K(p(t)) + V(q(t)) \tag{S1}$$

where K is the kinetic energy function and V is the potential energy function. The canonical variables $p(t)$ and $q(t)$ are grouped in the vector $z(t) = (q(t), p(t))$, so that their time evolution is described compactly using the Poisson bracket formalism

$$\dot{z} = \{H, z\} \tag{S2}$$

The Poisson bracket is defined as $\{H, z\} = \frac{\partial H}{\partial q}\frac{\partial z}{\partial p} - \frac{\partial H}{\partial p}\frac{\partial z}{\partial q}$ and the derivatives of z define the base vectors in phase space, that is $\frac{\partial z}{\partial p} = (0, 1)$ and $\frac{\partial z}{\partial q} = (1, 0)$.

Following Rangarajan, Dragt and Neri[1], we define the Lie operator $\{H, \cdot\}$, whose effect is the substitution of $\cdot$ with the function that it is applied to and evaluated accordingly. For instance, $\{H, \cdot\} f(a) = \{H, f\}(a)$. Thus, we can solve Eq. S2 in terms of z and t, for $t = \tau$ with initial condition $t = 0$:

$$z(\tau) = e^{-\tau\{H,\cdot\}} z(0) \tag{S3}$$

By definition $e^{-\tau\{H,\cdot\}} = 1 + \tau\{H, \cdot\} + \frac{\tau^2}{2!}\{H, \cdot\}^2 + \ldots$ and it is a sum of sequences of Poisson brackets which guarantees to provide a symplectic transformation. $e^{-\tau\{H,\cdot\}}$ is called a Lie transformation and corresponds to the Liouville formulation of the classical time-evolution operator.

A numerical scheme can be derived from Eq. S3 by building a symplectic map $\mathcal{M}$ that is a nonlinear transformation approximating $e^{-\tau\{H,\cdot\}}$. The symplectic map is defined as

$$\mathcal{M}_n = e^{-\tau\{H_n,\cdot\}} e^{-\tau\{H_{n-1},\cdot\}} \dots e^{-\tau\{H_1,\cdot\}} \tag{S4}$$

where H_i $(i = 1, \dots n)$ are partial Hamiltonians that sum up to H. Notice that Eq. S4 can generate a symplectic map for any truncation (for any integer n). In fact, each of the terms in Eq. S4 is a Lie transformation[1]. A practical choice for the partial Hamiltonians is

$$\mathcal{M}_k = e^{-\tau b_k\{V,\cdot\}} e^{-\tau a_k\{K,\cdot\}} \dots e^{-\tau b_1\{V,\cdot\}} e^{-\tau a_1\{K,\cdot\}}. \tag{S5}$$

with the constraints $\sum_i^n a_i = \sum_i^n b_i = 1$. Eq. S5 implies that a free system is evolved with kinetic energy K for $a_1\tau$ time, then a bound system is evolved with potential V and zero kinetic energy for $b_1\tau$, then again a free system for $a_2\tau$ time and so on.

Yoshida[2] has shown that it is possible to express the second order symplectic map $\mathcal{M}_2$ (that is, the Symplectic Leapfrog algorithm) by using twice the Baker-Campbell-Hausdorff formula for non-commutative operators. Furthermore, if the integrator is time-reverible, only the coefficients of the even powers of τ are non-zero[2]. Then Yoshida showed how to derive 4th and higher even order integrators as the symmetric product (or "triple jump" as referred by Tao[3]) of the second order symplectic maps. This approach leads to a series of simple polynomial equations with unique real solutions. However, not all the solutions of the symplectic map can be derived with this elegant approach. For the traditional derivation of the symplectic map, we need to apply all the operators in Eq. S5 to $z(0)$, starting from the rightmost operator and comparing the resulting expression to the Taylor series of $z(\tau)$ at a given order. For instance, the application of the two rightmost operators in the symplectic map gives a complex expression, in which the potential (blue) and kinetic (green) parts of the Lie operator are mixed:

$$z(\tau) = \mathcal{M} z(0) \tag{S6}$$

$$= \dots e^{-\tau b_1\{V,\cdot\}} e^{-\tau a_1\{K,\cdot\}} z(0) \tag{S7}$$

$$= \dots (1 - a_1\tau\{K,\cdot\} - b_1\tau\{V, 1 - a_1\tau\{K,\cdot\}\}) z(0) \tag{S8}$$

The $z(\tau)$ vector can then be written in two components as

$$
\begin{aligned}
p(\tau) &= p(0) - b_1\tau\frac{\partial V}{\partial q}(0) \\
q(\tau) &= q(0) + a_1\tau\frac{\partial K}{\partial p}(0) - b_1 a_1 \tau^2 \frac{\partial V}{\partial q}(0). \\
&= q(0) + a_1\tau p(\tau)
\end{aligned}
$$

For the two equations to be accurate we compare them with the Taylor series in the time domain, and find out that the optimal coefficients are $a_1 = 1$ and $b_1 = 1$. The resulting integrator corresponds to the semi-implicit Euler method (or Symplectic Euler). A similar analysis can be carried out for the second order map $\mathcal{M}_{k=2}$, which provides the following equations:

$$
\begin{aligned}
p(\tau) &= p(0) - b_1\tau\frac{\partial V}{\partial q}(0) - b_2\tau\frac{\partial V}{\partial q}\left(q(0) + a_1\tau p(0) - a_1 b_1\tau^2\frac{\partial V}{\partial q}(0)\right) \\
q(\tau) &= q(0) + (a_1 + a_2)\,p(0)\tau - (a_1 + a_2)\,b_1\tau^2\frac{\partial V}{\partial q}(0) \\
&\quad - a_2 b_2 \tau^2 \frac{\partial V}{\partial q}\left(q(0) + a_1\tau p(0) - a_1 b_1 \tau^2 \frac{\partial V}{\partial q}(0)\right).
\end{aligned}
\tag{S9}
$$

Here, however, the forces are computed at displaced positions (or at different times), hence we expand the forces in powers of q around $q(0)$. Comparing the expanded version of Eq. S9 to the Taylor series, up to the second order, one gets the following system of equations for the coefficients:

$$
\begin{cases}
a_1 + a_2 = 1 \\
b_1 + b_2 = 1 \\
a_1 b_2 = \dfrac{1}{2} \\
a_1 b_1 + a_2 b_1 + a_2 b_2 = \dfrac{1}{2}.
\end{cases}
\tag{S10}
$$

This system is undetermined. However, the simplest and most famous solution is $a_1 = 1, a_2 = 0, b_1 = 1/2, b_2 = 1/2$. Another solution is $a_1 = 1/2, a_2 = 1/2, b_1 = 0, b_2 = 1$ and this is the symplectic leapfrog or explicit Verlet method. Other, more accurate solutions are obtained by adding constraints to the third order terms. By constraining $a_1 b_1 b_2 = 1/6$, one obtains an integrator that is exact to 3rd order in the momentum and it has an error equal to $(1/24)p_0\tau^3\partial^2 V(0)/\partial q^2$ on the position. Such integrator has the coefficients $a_1 = 3/4, a_2 = 1/4, b_1 = 1/3, b_2 = 2/3$ and it is not time-reversible. Higher order integrators can

be obtained with the same procedure[1,4]. For the fourth order map $\mathcal{M}_{n=4}$, we can derive and solve the system of equations with the help of the SageMath[5] computer algebra system:

$$\begin{cases} a_1 + a_2 + a_3 + a_4 = 1 \\ b_1 + b_2 + b_3 + b_4 = 1 \\ (a_1 + a_2 + a_3 + a_4)b_1 + (a_2 + a_3 + a_4)b_2 + (a_3 + a_4)b_3 + a_4 b_4 = \frac{1}{2} \\ a_1 b_2 + (a_1 + a_2)b_3 + (a_1 + a_2 + a_3)b_4 = \frac{1}{2} \\ (a_1 + a_2 + a_3)a_4 b_4 + (a_1 a_2 + a_1 a_3 + a_1 a_4)b_2 + ((a_1 + a_2)a_3 + (a_1 + a_2)a_4)b_3 = \frac{1}{6} \\ a_1 b_1 b_2 + ((a_1 + a_2)b_1 + a_2 b_2)b_3 + ((a_1 + a_2 + a_3)b_1 + (a_2 + a_3)b_2 + a_3 b_3)b_4 = \frac{1}{6} \\ \frac{1}{2}a_1^2 b_2 + \frac{1}{2}(a_1^2 + 2a_1 a_2 + a_2^2)b_3 + \frac{1}{2}(a_1^2 + 2a_1 a_2 + a_2^2 + 2(a_1 + a_2)a_3 + a_3^2)b_4 = \frac{1}{6} \\ (a_1 a_2 + a_1 a_3 + a_1 a_4)b_1 b_2 + (((a_1 + a_2)a_3 + (a_1 + a_2)a_4)b_1 + (a_2 a_3 + a_2 a_4)b_2)b_3 + \\ \qquad +((a_1 + a_2 + a_3)a_4 b_1 + (a_2 + a_3)a_4 b_2 + a_3 a_4 b_3)b_4 = \frac{1}{24} \\ \frac{1}{2}(a_1^2 + 2a_1 a_2 + a_2^2 + 2(a_1 + a_2)a_3 + a_3^2)a_4 b_4 + \frac{1}{2}(a_1^2 a_2 + a_1^2 a_3 + a_1^2 a_4)b_2 + \\ \qquad +\frac{1}{2}((a_1^2 + 2a_1 a_2 + a_2^2)a_3 + (a_1^2 + 2a_1 a_2 + a_2^2)a_4)b_3 = \frac{1}{24} \\ a_1^2 b_1 b_2 + ((a_1^2 + 2a_1 a_2 + a_2^2)b_1 + (a_1 a_2 + a_2^2)b_2)b_3 + ((a_1^2 + 2a_1 a_2 + a_2^2 + 2(a_1 + a_2)a_3 + a_3^2)b_1 + \\ \qquad +(a_1 a_2 + a_2^2 + (a_1 + 2a_2)a_3 + a_3^2)b_2 + ((a_1 + a_2)a_3 + a_3^2)b_3)b_4 = \frac{1}{8} \\ \frac{1}{6}a_1^3 b_2 + \frac{1}{6}(a_1^3 + 3a_1^2 a_2 + 3a_1 a_2^2 + a_2^3)b_3 + \frac{1}{6}(a_1^3 + 3a_1^2 a_2 + 3a_1 a_2^2 + a_2^3 + 3(a_1 + a_2)a_3^2 + a_3^3 + \\ \qquad +3(a_1^2 + 2a_1 a_2 + a_2^2)a_3)b_4 = \frac{1}{24} \\ a_1 a_2 b_2 b_3 + ((a_1 + a_2)a_3 b_3 + (a_1 a_2 + a_1 a_3)b_2)b_4 = \frac{1}{24} \end{cases} \tag{S11}$$

It is important to notice that this system is undetermined. We need to choose another constraint to effectively get numerical values for a_k and b_k coefficients. A reasonable choice to saturate the system is to set $b_1 = 0$, which leads to three possible set of real coefficients: the first set was first published by Forest and Ruth[6], and then derived by Candy and

Rozmus[7],

$$\begin{cases} a_1 &= \frac{2^{1/3}+2^{-1/3}+2}{6} \\ a_2 &= -\frac{2^{1/3}+2^{-1/3}-1}{6} \\ a_3 &= -\frac{2^{1/3}+2^{-1/3}-1}{6} \\ a_4 &= \frac{2^{1/3}+2^{-1/3}+2}{6} \\ b_1 &= 0 \\ b_2 &= \frac{2^{4/3}+2^{2/3}+4}{6} \\ b_3 &= -\frac{2^{7/3}+2^{5/3}+2}{6} \\ b_4 &= \frac{2^{4/3}+2^{2/3}+4}{6} \end{cases}, \tag{S12}$$

while the second was reported by Brewer *et al.*[8],

$$\begin{cases} a_1 &= \sqrt{3}/6+1/2 \\ a_2 &= -\sqrt{3}/3 \\ a_3 &= \sqrt{3}/3 \\ a_4 &= -\sqrt{3}/6+1/2 \\ b_1 &= 0 \\ b_2 &= -\sqrt{3}/6+1/4 \\ b_3 &= 1/2 \\ b_4 &= \sqrt{3}/6+1/4 \end{cases} \tag{S13}$$

and a third set that is equal to the set in Eq. S13 but with b_2 swapped with b_4, a_2 swapped with a_3, and a_1 swapped with a_4. These three are the only real solutions that we have found to the system in Eq. S11 with the added constraint $b_1 = 0$. There are other complex solutions that we disregarded. Another famous choice for the saturating conditions is $a_4 = 0$ and $b_1 = b_4$, which leads to the 4th order integrator allegedly discovered by Neri and re-derived by Yoshida[2] and by Forest and Ruth[6].

B. Details about the decoupling symplectic integration algorithm

In this section we use the results of derivation we have described in section I A to carry out a symplectic map for the integration of the pair-decoupled system. We define the

square matrix A which has the same dimensionality of the Hessian matrix (in whatever coordinate system is used for the decoupling), and write the pair decoupled Hessian matrix as $A \otimes \partial^2 V/\partial q^2$, where $\otimes$ is the direct product, i.e. given the matrix B and C of same sizes $(B \otimes C)_{ij} = B_{ij} C_{ij}$. A is defined such that $A_{ii} = 1$ and $A_{i \neq j} \in [0, 1]$. The matrix A can be interpreted as the adjacency matrix of the graph of the couplings, i.e. if no decoupling is applied, then A is the adjacency matrix of a complete simple undirected graph. Instead, when the decoupling is applied, some of the edges are weighted by α (or absent if $\alpha = 0$).

Let us define a pair-decoupling operator $\hat{D}_\alpha$, which operates on the potential and transforms it into the pair-decoupled potential $\hat{D}_\alpha V = \tilde{V}$. Now we can write the time propagation for the momentum variable as

$$\tilde{p}(t + b_k \tau) = e^{-\tau b_k \hat{D}_\alpha \{V, \cdot\}} p(t) = e^{-\tau b_k \{\tilde{V}, \cdot\}} p(t) \tag{S14}$$

$$= p(t) - \tau b_k \hat{D}_\alpha \{V, p\}(t) \tag{S15}$$

$$= p(t) - \tau b_k \hat{D}_\alpha \left[-\frac{\partial V}{\partial q} \right](t) = p(t) - \tau b_k \left[-\frac{\partial \tilde{V}}{\partial q}(t) \right] \tag{S16}$$

$$\approx p(t) - \tau b_k \hat{D}_\alpha \left[-\frac{\partial V}{\partial q}(t - c_{k-1}\tau) - \frac{d}{dt}\left(\frac{\partial V}{\partial q} \right)(t) c_k \tau \right] \tag{S17}$$

$$= p(t) - \tau b_k \left[-\frac{\partial \tilde{V}}{\partial q}(t - c_{k-1}\tau) - \frac{\partial^2 \tilde{V}}{\partial q^2}(t) \dot{q}(t) c_k \tau \right] \tag{S18}$$

$$= p(t) - \tau b_k \left[-\frac{\partial \tilde{V}}{\partial q}(t - c_{k-1}\tau) - \left(A \otimes \frac{\partial^2 V}{\partial q^2}(t) \right) \dot{q}(t) c_k \tau \right]. \tag{S19}$$

In step S17 we linearized the force $-\partial V/\partial q$ around the time $t - c_{k-1}\tau$, where the c_k are real parameters to be optimized, so that the canonical variables are equal to their corresponding time Taylor expansion up to a given order of τ. Because of this linearization, we say that the force is updated with a local harmonic approximation. Also note that in step S17 the derivative of the force is computed at time t and not $t - c_{k-1}\tau$ as expected. This is because we implicitly used the auxiliary position variable q_{aux}, which is the best approximation for $q(t)$. In step S18 we used the abstract definition $\hat{D}_\alpha V = \tilde{V}$, and in the last step we used the definition we gave of pair-decoupling, that is $\left[\partial^2 \tilde{V}/\partial q^2\right]_{ij} = \alpha \left[\partial^2 V/\partial q^2\right]_{ij}$. In Eq. S19 the pair decoupled force $-\partial \tilde{V}/\partial q$ is evaluated at the previous time $t - c_{k-1}\tau$, and comparing Eq. S19 with the rightmost hand-side of S16, the reader sees that the pair decoupled force at time t corresponds to $-\frac{\partial \tilde{V}}{\partial q}(t) \approx \left[-\frac{\partial \tilde{V}}{\partial q}(t - c_{k-1}\tau) - \left(A \otimes \frac{\partial^2 V}{\partial q^2}(t) \right) \dot{q}(t) c_k \tau \right]$. For practical purposes we can write this expression as $\tilde{F}_k = \tilde{F}_{k-1} - c_k \tau \partial^2 \tilde{V}/\partial q^2 \cdot \tilde{p}_k/m$. Thus, given an

initial value of the pair decoupled force $\tilde{F}_0$, we can propagate it in time.

We can now write the map

$$\mathcal{M}_n \approx e^{-\tau b_n \hat{D}\{V,\cdot\}} e^{-\tau a_n \{K,\cdot\}} \dots e^{-\tau b_1 \hat{D}\{V,\cdot\}} e^{-\tau a_1 \{K,\cdot\}}, \tag{S20}$$

that depends on the additional c_k coefficients and α. The actual algorithm to update the forces is just a loop over the index k of the following 4 steps.

$$\tilde{p}_k = \tilde{p}_{k-1} + b_k \tau \tilde{F}_{k-1} \tag{S21}$$

$$\tilde{q}_k = \tilde{q}_{k-1} + a_k \tau \tilde{p}_k / m \tag{S22}$$

$$\tilde{q}_{aux} = \tilde{q}_k + \tilde{p}_k \tau \sum_j^k (b_j - a_j)/m \tag{S23}$$

$$\tilde{F}_k = \tilde{F}_{k-1} - c_k \tau \frac{\partial^2 \tilde{V}(q_{k,aux})}{\partial \tilde{q}^2} \cdot \tilde{p}_k / m, \tag{S24}$$

where $\tilde{q}_k = \tilde{q}\left(\tau \sum_j^k a_j\right)$, $\tilde{p}_k = \tilde{p}\left(\tau \sum_j^k b_j\right)$ and $\tilde{F}_k = \tilde{F}\left(\tau \sum_j^k c_j\right)$. Notice that the force update requires all the variables: force, position and momentum. The last two must be at the same time t. This is the reason why we introduce the ausiliary variable $\tilde{q}_{aux}$, which brings the ausiliary position forward (or backward) in time to match the momentum. In this way the force variable $\tilde{F}(t)$ is integrated alongside $\tilde{q}(t)$ and $\tilde{p}(t)$, on the same footing. However, one should bear in mind that α influences only how the force is updated. Therefore it is necessary to give the initial value of $\tilde{F}$ as an input to the algorithm. A good choice would be to initiate the simulation at a potential minimum, assuming that the pair-decoupled force is zero, just like the normal force. In case another initial condition is chosen, the system would behave as if the pair decoupling were applied when the simulation begins.

To determine the c_k coefficients we focus on the case $\alpha = 1$ (which implies $\tilde{q} \approx q$, $\tilde{p} \approx p$, and $\tilde{F} \approx F$). The reader might have noticed that, for $\tilde{F}_n$ to be most accurate, $c_k \approx a_k$, because $-\tilde{F}_n$ should approximate the gradient at coordinate q_n. This observation is correct for the harmonic oscillator, for which Eq. S24 matches the exact Taylor series of the force. However, for non-harmonic potentials we found that this is not always the case. In fact, other coefficients might be more appropriate. The reason is that with Eq. S24 we neglect the contributions coming from the potential derivatives with order higher than 2, thus we necessarily introduce a small error that we do not want to accumulate during the simulation.

The map in Eq. S20 can be solved for the desired order of k by comparing the position, momentum and force variables with their Taylor expansion, in the same way as we did in section I A. The second order ($k = 2$) solution is given by the coefficients

$$\begin{cases} a_1 &= \frac{1}{2} \\ a_2 &= \frac{1}{2} \\ b_1 &= 0 \\ b_2 &= 1 \\ c_1 &= \frac{1}{2} \\ c_2 &= \frac{1}{2} \end{cases} \tag{S25}$$

which corresponds to a Leapfrog algorithm that runs on a locally harmonic potential. Notice that, since we enforce $c_n = a_n$, this solution does not introduce further approximations for harmonic potentials. In fact, for harmonic or bilinear potentials, the dynamics would be indistinguishable from a standard symplectic leapfrog.

Since the position and momentum update operators are not influenced by the force update operator, we can use the set of coefficients $\{a_k\}$ and $\{b_k\}$ we derived in section I A. In particular, we tried both the solution in Eq. S12 (also reported in Ref. 6), and the solution in Eq. S13 (also reported in Ref. 8). Once the $\{a_k\}$ and $\{b_k\}$ are established, we derive the $\{c_k\}$ coefficients to get the 4th order map. Enforcing the coefficients in Eq. S12 is straightforward and the optimal solution is unambiguously $\{c_k\} = \{a_k\}$. This is our first choice. On the other hand, enforcing the coefficients in Eq. ,S13 which are usually superior in terms of energy conservation for molecular dynamics, we find that it is possible to derive an algorithm that is accurate to the second order in τ with respect to position, momentum and force. If we enforce a third order accuracy on the momentum, we get:

$$\begin{aligned} c_1 &= c_1 \\ c_2 &= -\frac{2}{23}(12\sqrt{3} + 31)c_1 + \frac{28}{69}\sqrt{3} + \frac{19}{23} \\ c_3 &= -2c_1(4\sqrt{3} + 7) + \frac{4}{3}\sqrt{3} + 3 \\ c_4 &= \frac{1}{23}c_1(208\sqrt{3} + 361) - \frac{40}{23}\sqrt{3} - \frac{65}{23}, \end{aligned}$$

and if we enforce the third order accuracy on the position, we get

$$
\begin{aligned}
c_1 &= c_1 \\
c_2 &= -c_1 + \frac{1}{6}\sqrt{3} + \frac{1}{2} \\
c_3 &= c_1(4\sqrt{3} + 7) - \frac{7}{6}\sqrt{3} - \frac{3}{2} \\
c_4 &= -c_1(4\sqrt{3} + 7) + \sqrt{3} + 2.
\end{aligned}
$$

The system is undetermined by one degree of freedom in either case.

There are at least five options to approach this problem. The first option is to enforce accuracy up to the third order for both $q(\tau)$ and $p(\tau)$. In this case we obtain a saturated system that is actually accurate to the *fourth* order, with coefficients:

$$
\begin{cases} c_k & = a_k \cdot \end{cases} \tag{S26}
$$

Although this solution looks appealing, the integrator is not time-reversible and it works well only for harmonic or bilinear potentials. In the harmonic and bilinear potential cases and $c_k = a_K$, Eq. S24 is not an approximation, and the integrator is as accurate as the one in Ref. 8.

The second option is to enforce third order accuracy in $p(\tau)$ and $c_1 = c_4$.

$$
\begin{cases}
c_1 & = \frac{5}{26} \\
c_2 & = \frac{8}{39}\sqrt{3} + \frac{4}{13} \\
c_3 & = -\frac{8}{39}\sqrt{3} + \frac{4}{13} \\
c_4 & = \frac{5}{26}
\end{cases} \tag{S27}
$$

The third option is to enforce third order accuracy in $p(\tau)$ and $c_1 = -c_4$

$$
\begin{cases}
c_1 & = \frac{5}{48}\sqrt{3} \\
c_2 & = \frac{1}{8}\sqrt{3} + \frac{1}{2} \\
c_3 & = -\frac{1}{8}\sqrt{3} + \frac{1}{2} \\
c_4 & = -\frac{5}{48}\sqrt{3}
\end{cases} \tag{S28}
$$

The fourth option is to enforce third order accuracy in $q(\tau)$ and $c_1 = c_4$:

$$\begin{cases} c_1 &= \frac{1}{4} \\ c_2 &= \frac{1}{6}\sqrt{3} + \frac{1}{4} \\ c_3 &= -\frac{1}{6}\sqrt{3} + \frac{1}{4} \\ c_4 &= \frac{1}{4} \end{cases} \tag{S29}$$

And finally the fifth option is to enforce third order accuracy in $q(\tau)$ and $c_1 = -c_4$:

$$\begin{cases} c_1 &= \frac{1}{6}\sqrt{3} \\ c_2 &= \frac{1}{2} \\ c_3 &= \frac{1}{2} \\ c_4 &= -\frac{1}{6}\sqrt{3} \end{cases} \tag{S30}$$

Apart from the first otpion, all solutions are stable enough for general potentials, in the sense that they conserve the total energy over time, with energy fluctuations that are comparable to second order methods, such as the Velocity-Verlet[9] or Symplectic Leapfrog methods. However, we discourage to use the fourth and the fifth options, because they produce greater errors, at least for rigid systems.

We call this kind of integration rule the Symplectic Explicit with Force integration (SEF) algorithm. If the second order ($k = 2$) scheme is used, the coefficients are reported in Eq.S25, and we call it “SEF2”. If one of the fourth order schemes is used, we call it “SEF4”.

C. Evolution of the pair-decoupled Monodromy matrix

The SEF algorithm can be easily modified to integrate also the monodromy matrix in a similar fashion of what is described in the Appendix of Ref. 8. The modified algorithm

consists of the following steps iterated twice (for SEF2) or four times (for SEF4):

$$\tilde{p}_k = \tilde{p}_{k-1} + b_k \tau \tilde{F}_{k-1}$$
$$\tilde{q}_k = \tilde{q}_{k-1} + a_k \tau \tilde{p}_k$$

$$q_{tmp} = \tilde{q}_k + \tilde{p}_k \tau \sum_i^k (b_i - a_i)$$
$$\tilde{h} = \frac{\partial^2 \tilde{V}(q_{tmp})}{\partial \tilde{q}^2}$$
$$\tilde{F}_k = \tilde{F}_{k-1} - c_k \tau \tilde{h} \cdot \tilde{p}_k$$

$$\tilde{M}_{pp,k} = \tilde{M}_{pp,k-1} - b_k \tau \tilde{h} \cdot \tilde{M}_{qp,k-1}$$
$$\tilde{M}_{pq,k} = \tilde{M}_{pq,k-1} - b_k \tau \tilde{h} \cdot \tilde{M}_{qq,k-1}$$
$$\tilde{M}_{qp,k} = \tilde{M}_{qp,k-1} + a_k \tau \tilde{M}_{pp,k}$$
$$\tilde{M}_{qq,k} = \tilde{M}_{qq,k-1} + a_k \tau \tilde{M}_{qp,k},$$

where $\tilde{M}_{pp} = \partial\tilde{p}/\partial p_0$, $\tilde{M}_{pq} = \partial\tilde{p}/\partial q_0$, $\tilde{M}_{qp} = \partial\tilde{q}/\partial p_0$, $\tilde{M}_{qq} = \partial\tilde{q}/\partial q_0$ are four square blocks of the monodromy matrix. $\tilde{M}(0)$ is initialized equal to the canonical symplectic matrix $\mathcal{J}$. Notice that the only information required for the evolution of $\tilde{M}$ is encoded in the pair-decoupled Hessian matrix $\tilde{h}$. In fact, a_k, b_k and τ are input parameters of the simulation. Hence, $\tilde{M}$ is the pair-decoupled monodromy matrix. In Figure S1, we show that SEF2 and SEF4 algorithms preserve the properties of $\Upsilon(t)$ and $\tau(t)$ for the H_2O molecule even when the pair of H atoms are decoupled with $\alpha = 0$.

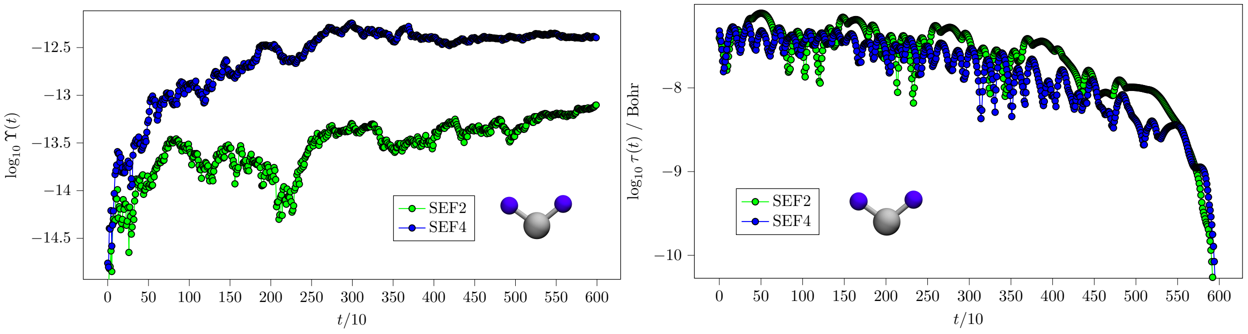

FIG. S1. $\Upsilon(t)$ and $\tau(t)$ for the water molecule with H−H decoupling with $\alpha = 0$. A cartoon of the water molecule is depicted in the bottom part of both graphs with blue colored H atoms to indicate that the H atoms are decoupled. This picture should be considered in comparison with Fig. 2 of the paper

As a proof that the decoupled degrees of freedom are indeed not coupled, we show that the Monodromy matrix can be factorized as a two block matrix, where each block preserves Symplectic symmetry. One block of the matrix spans the bending mode and the other the two stretching modes. This is displayed in Fig. S2, where $\Upsilon(t)$ is shown to be close to 0 at all times for both the subsystems along the simulation evolved with SEF4.

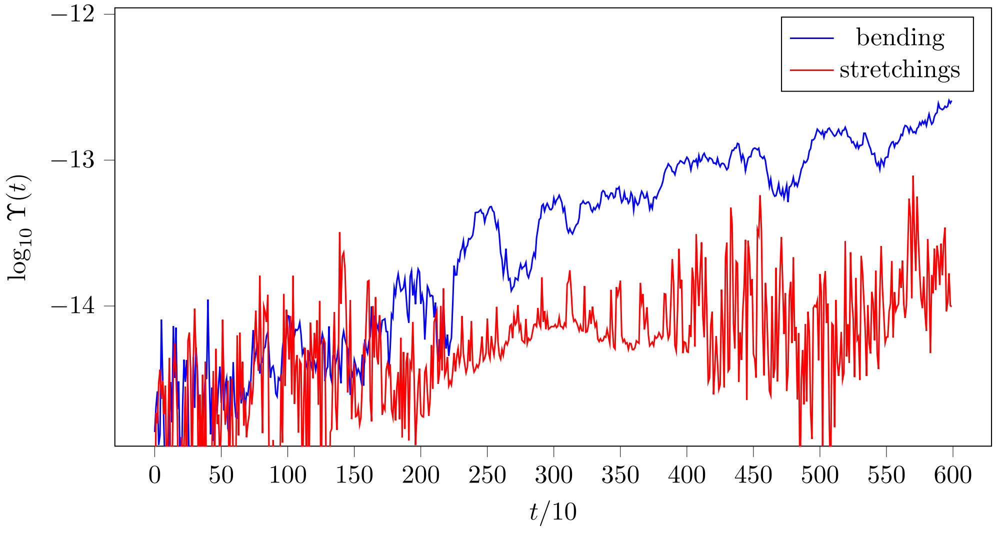

FIG. S2. $\log_{10} \Upsilon(t)$ for the bending and stretching modes of the water molecule

REFERENCES

[1]Govindan Rangarajan, F Neri, and AJ Dragt. Solvable map representation of a nonlinear symplectic map. *Part. Accel.*, 28:119–124, 1990. I A, I A, I A

[2]Haruo Yoshida. Construction of higher order symplectic integrators. *Physics letters A*, 150(5-7):262–268, 1990. I A, I A, I A

[3]Molei Tao. Explicit symplectic approximation of nonseparable hamiltonians: Algorithm and long time performance. *Physical Review E*, 94(4):043303, 2016. I A

[4]AJ Dragt, Filippo Neri, Govindan Rangarajan, David R Douglas, Liam M Healy, and Robert D Ryne. Lie algebraic treatment of linear and nonlinear beam dynamics. *Annual Review of Nuclear and Particle Science*, 38(1):455–496, 1988. I A

[5]The Sage Developers. *SageMath, the Sage Mathematics Software System (Version 9.5)*, 2022. `https://www.sagemath.org`. I A

[6]Etienne Forest and Ronald D Ruth. Fourth-order symplectic integration. *Physica D: Nonlinear Phenomena*, 43(1):105–117, 1990. I A, I A, I B

[7]J Candy and W Rozmus. A symplectic integration algorithm for separable hamiltonian functions. *Journal of Computational Physics*, 92(1):230–256, 1991. I A

[8]Mark L Brewer, Jeremy S Hulme, and David E Manolopoulos. Semiclassical dynamics in up to 15 coupled vibrational degrees of freedom. *J. Chem. Phys.*, 106(12):4832–4839, 1997. I A, I B, I B, I C

[9]Loup Verlet. Computer "experiments" on classical fluids. i. thermodynamical properties of lennard-jones molecules. *Physical review*, 159(1):98, 1967. I B
